# Rafts of change: microbial and functional dynamics in simulated *Sargassum* strandings

**DOI:** 10.1128/aem.02357-25

**Published:** 2026-03-31

**Authors:** Shrinivas Nandi, Timothy G. Stephens, Rebecca Garcia, Mayra Sánchez-García, Loretta M. Roberson, Jose L. Avalos, Shishir P. S. Chundawat, Debashish Bhattacharya

**Affiliations:** 1 Department of Biochemistry and Microbiology, Rutgers University, New Brunswick, New Jersey, USA; 2 Department of Chemical and Biochemical Engineering, Rutgers University 547644, Piscataway, New Jersey, USA; 3 Marine Biological Laboratory 42700, Woods Hole, Massachusetts, USA; 4 Department of Chemical and Biological Engineering, The Omenn-Darling Bioengineering Institute, The Andlinger Center for Energy and the Environment, and the High Meadows Environmental Institute of Princeton University, Princeton, New Jersey, USA; University of Delaware, Lewes, Delaware, USA

**Keywords:** microbiome, multi-omics, *Sargassum*, seaweed degradation

## Abstract

Massive influxes of pelagic *Sargassum* spp. across the tropical Atlantic and Caribbean regions have created urgent ecological and economic challenges that need to be addressed to stabilize local ecosystems. Use of this abundant biomass feedstock resource for biorefining and bioproducts manufacturing is a promising avenue, but this goal requires elucidating the microbial processes that regulate *Sargassum* degradation, which are still poorly understood. Here, we investigated the microbial degradation of the benthic *Sargassum filipendula* by native microbiota using multi-omics approaches. Metagenomic and meta-transcriptomic analyses identified diverse carbohydrate-active enzymes (CAZymes), including alginate lyases, fucoidanases, and cellulases, that were differentially expressed over the course of the *in vitro* degradation timeline. Furthermore, we identified the need for arsenic detoxification pathways in microbes utilizing *Sargassum*-derived substrates. We observed a suite of factors influencing microbial dynamics, including prokaryotic competition, arsenic detoxification, viruses, and substrate availability. Lineages potentially capable of degrading recalcitrant polysaccharides such as fucoidan appeared to be rapidly outcompeted by other bacteria that utilized simpler substrates like mannitol. These results highlight the metabolic potential of native marine microbial communities to degrade complex *Sargassum* polysaccharides and the importance of the *in vitro* degradation experiment time scale to capture the activities of non-dominant specialists. Our findings elucidate microbial ecosystem dynamics during *Sargassum* degradation and provide novel insights that can be used to advance the development of biotechnological approaches that leverage renewable *Sargassum* biomass as a biorefinery feedstock of the future.

**IMPORTANCE:**

This work addresses a crisis in the tropical Atlantic and Caribbean regions, the massive population growth and stranding of the floating brown seaweed *Sargassum*, which is wreaking havoc on ecosystems and fouling beaches vital to local tourism. One solution to this problem is to utilize the seaweed as feedstock to generate useful bioproducts. This approach requires characterizing the microbiome of *Sargassum* that drives its degradation in nature. To this end, we devised an in-lab degradation assay using *Sargassum* and identified a variety of carbohydrate-active enzymes, including alginate lyases, fucoidanases, and cellulases which break down seaweed cell wall polysaccharides. We also find that microbes compete in the closed reactors, with diversity being reduced over time. These results highlight the metabolic potential of native marine microbial communities to degrade *Sargassum* and elucidate microbial ecosystem dynamics during this process. These insights allow the use of renewable *Sargassum* as a biorefinery feedstock of the future.

## INTRODUCTION

In recent years, the unprecedented proliferation of *Sargassum* across the Atlantic has drawn global attention due to its extensive ecological, economic, and public health impacts. Since the formation of the Great Atlantic Sargassum Belt in 2011, blooms have increased in both frequency and intensity (1, 2), with stranded biomass approaching 35 million metric tons in 2025 (3). These massive tides of holopelagic brown algae, primarily composed of *S. natans* and *S. fluitans* strains, disrupt coastal ecosystems, shade coral reefs, dissuade tourism by restricting beach access (i.e., harming local economies), and pose health hazards through the release of toxic gases such as hydrogen sulfide and ammonia during decomposition (4–7). *Sargassum* can also sequester toxic heavy metals, such as arsenic, which presents additional health risks to humans and the surrounding ecosystems (7–10). Despite the challenges posed by *Sargassum* strandings, this macroalga holds great potential as a sustainable feedstock for biorefineries of the future. Seasonal blooms can be harvested in large volumes, providing an abundant yet underutilized biomass source rich in organic carbon, polysaccharides, and essential nutrients (11). Studies have explored the use of seaweed in producing methane and other biofuels (12), while others have investigated the feasibility of extracting high-value polysaccharides and rare-earth elements from *Sargassum* (13). As a fast-growing species, this seaweed also serves as a significant carbon sink, absorbing atmospheric CO_2_ (14).

Holopelagic *Sargassum* drifts across diverse environments throughout its life cycle, creating a flexible and open symbiotic system that supports constant microbial turnover and recruitment (15). Composition of the *Sargassum* microbiome varies significantly in the different tissue types (e.g., frond vs stipe) (16), seasons (17), geographic locations (18, 19), and growth cycles (20). Exudates released by *Sargassum*, combined with its mucus-rich surface, provide an ideal substrate for microbial colonization (15). Diverse biofilms have been observed on *Sargassum*, often enriched in phototrophs, heterotrophs, organic matter degraders, and diazotrophs, particularly members of the Rhodobacteraceae and Cyanobacteria (15, 18, 21–23). A recurring feature of the *Sargassum* microbiome is the high prevalence of *Vibrio* spp., which are frequently enriched relative to ambient seawater, particularly under high-exudate conditions offshore (23–25). These biofilms and endophytic bacteria form mutualistic relationships with the macroalgal host (15, 22, 26). For example, nitrogen-fixing heterotrophs may supply biologically available nitrogen in otherwise oligotrophic environments (21). Similarly, the microbiome contributes to phosphorus and sulfur cycling, and the microbial community benefits from access to nutrient-rich macroalgal exudates (15).

When large rafts of *Sargassum* wash ashore during stranding events, marked shifts in microbial community composition are observed, likely tied to the decomposition of the stranded macroalgae (23, 27, 28). These events are typically associated with a decline in overall microbial diversity and the emergence of dominant taxa such as Vibrionales, Alteromonadales, Oceanospirillales, and Rhodobacteraceae (19, 23, 24, 29, 30). The high abundance of Vibrionales is of concern, due to the presence of potentially pathogenic species (27). Furthermore, these microbes can introduce novel pathogens to otherwise already vulnerable ecosystems, for example, coral reefs in the Caribbean, which are affected by different diseases (31, 32). The degradation of *Sargassum* poses a significant challenge, with millions of dollars spent annually on its removal from beaches and shorelines (33). Its decomposition is inherently slow and complex due to the recalcitrant polysaccharide matrix in this seaweed, which includes fucoidan, alginate, cellulose, and laminarin (34, 35). Microbial taxa colonizing stranded *Sargassum* are frequently proposed as key agents in its breakdown, owing to their diverse repertoire of carbohydrate-active enzymes (CAZymes). Research has focused on identifying marine prokaryotes capable of degrading these polysaccharides, assaying surface-associated microbiota, free-living microbes in the surrounding water column, and the gut microbiomes of fish and isopods that feed on *Sargassum* (23, 36, 37).

Fucoidan-degrading enzymes (i.e., fucoidanases) cleave fucosidic linkages and include seven glycoside hydrolase (GH) families in the CAZy database: GH29, GH95, GH107, GH139, GH141, GH151, and GH168 (38, 39). For instance, *Lentimonas* spp. (Verrucomicrobiota) harbors a mega-plasmid encoding multiple GH-family CAZymes, including GH29, GH95, and GH141, enabling limited fucoidan degradation (40). In addition to GHs, sulfatases are essential for removing sulfate groups from fucoidan and fall into five major CAZy families: S1_13, S1_15, S1_16, S1_17, and S1_25. Alginate degradation (lyases) has also been well studied, and these enzymes are currently classified into 14 polysaccharide lyase (PL) families (38, 39). Cellulase-degrading enzymes have also been classified broadly into nine GH families and laminarin-degrading enzymes into 16 GH families (38).

Although previous studies have characterized *Sargassum*-associated microbial communities, primarily using 16S rRNA sequencing, fewer studies have performed a multi-omics analysis of degrading *Sargassum* (15). Whereas 16S rRNA-based profiling provides taxonomic composition, the metagenomic and metatranscriptomic data generated by our study are required to resolve the functional potential and active metabolic pathways of the microbiome. This approach allows analysis of processes such as polysaccharide degradation and novel enzyme discovery (41). In this study, we conducted a simulated *in vitro* degradation experiment using the benthic species *Sargassum filipendula*, which occurs from the Caribbean to Cape Cod, Massachusetts and has an overall carbohydrate composition similar to holopelagic *Sargassum* (42, 43). Using integrated metagenomics and meta-transcriptomics, we elucidated the shifts in microbial community structure and functional activity during its degradation by microbiota native to it, with the goal of identifying key microbial processes associated with the degradation of the more recalcitrant *Sargassum* polysaccharides.

## MATERIALS AND METHODS

### Experimental design

**Fig 1:**
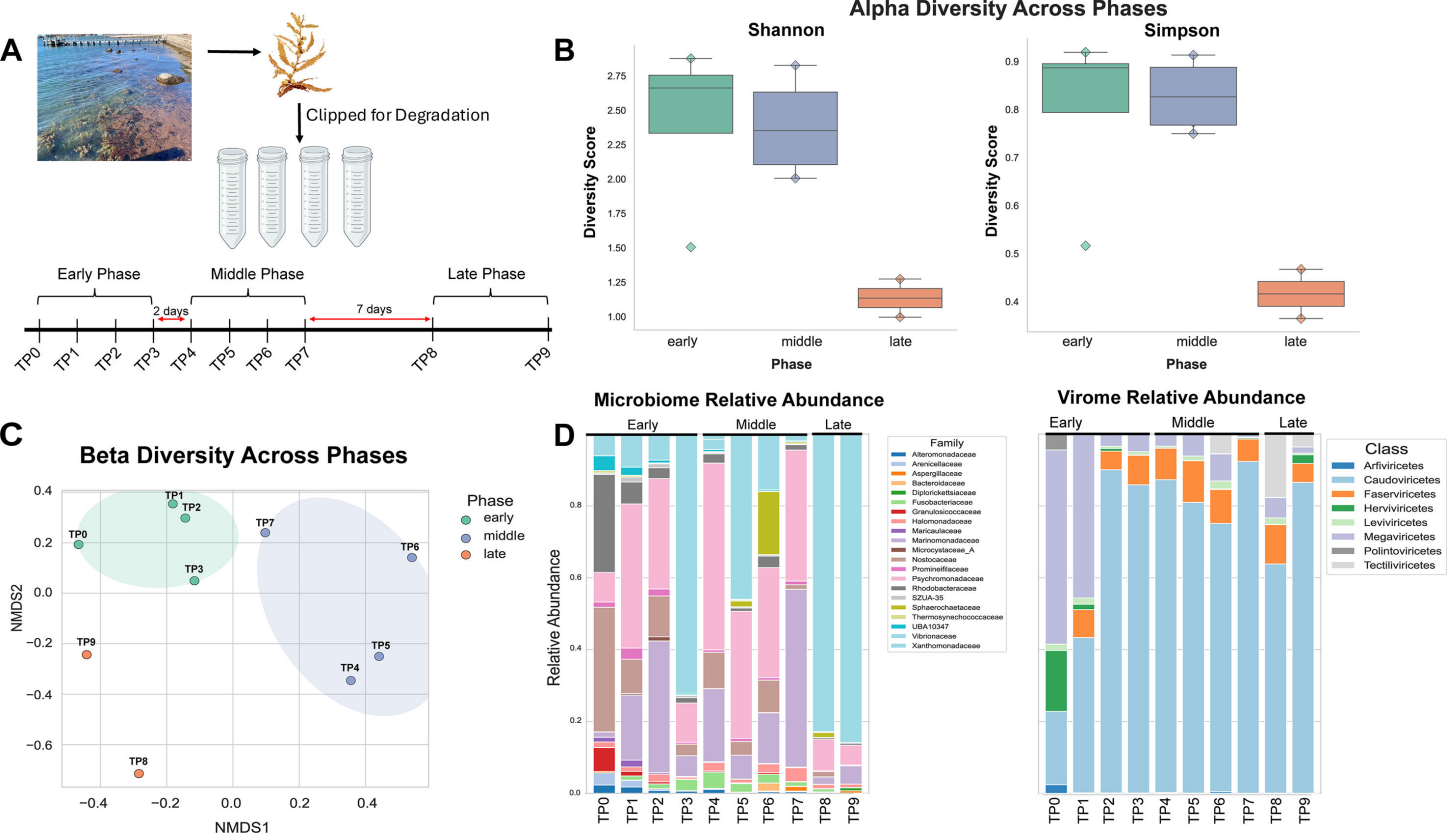
Microbiome dynamics over the course of the degradation experiment. (**A**) Experimental set up, showing the intertidal sampling site at Waterfront Park, Woods Hole, MA. One set of samples was clipped and immediately stored (fresh *Sargassum*). Phase distribution, that is, time points within each phase are shown. Red arrows indicate the time between each collection. (**B**) Alpha diversity metrics for the microbiome (eMAG and pMAG) are shown. The *y*-axis shows the diversity or evenness score, and the *x*-axis shows the phases, green: early phase, purple: middle phase, and orange: late phase. (**C**) Beta diversity (Aitchison distance) shown as an NMDS plot. The color scheme of points is shown in the legend and is consistent with image (**B**). Ellipses are drawn at 3.0 standard deviations from the mean. The time point of each point is noted in the figure. (**D**) Left: microbiome relative abundance (*y*-axis) is plotted. Time points have been presented on the *x*-axis and the top groups time points into respective phases. Taxa have been grouped at a family level annotation based on GTDBk-taxonomy and are presented in the legend. Right: the relative abundance of the virome shown at the class level (Baltimore classification).

About 1 kg of *S. filipendula* was collected from Waterfront Park, Woods Hole, MA (SFi1124-WH-S1, 41.5249°N, 70.6724°W) and shipped to Rutgers University in a cooler while wrapped in damp paper towels. *S. filipendula* was divided and placed into 64 (15 mL Falcon tubes), each tube containing ~5 g of intact macroalgae and 1 ml of autoclaved seawater (35 ppt). The top of each tube was fitted with a rubber balloon to prevent excessive buildup of gas from the degrading samples. Samples were stored in an incubator, exposed to a day-night cycle (16:8), at 38 µmol m^−2^ s^−1^ and a temperature of 22°C. Every other day for the first 2 weeks (days 1–14), four tubes were randomly collected and frozen in a −80°C freezer. After this initial 2-week period, two additional collections were performed at the end of each following week (i.e., on day 21 [week 3] and day 28 [week 4]). One sample (four replicates) was also collected on the day the macroalgae was received at Rutgers University, labeled time point 0 (TP0). A total of 10 time points were collected, labeled TP0–TP9 (Fig. 1A). DNA and RNA were extracted from each tube using the ZymoBIOMICS DNA/RNA kit, using the manufacturer’s protocol. Due to low DNA yields, all four replicates per time point were pooled into a single sample; RNA yields were adequate, and no pooling was required for sequencing. Samples were shipped to Azenta Life Sciences for 2 × 150 bp sequencing on the NovaSeq X Plus machine.

### Metagenome assembly

Metagenome-assembled genomes (MAGs) were constructed for prokaryotes, eukaryotes, plasmids, and viruses using the Naïve ATLAS workflow (https://github.com/TimothyStephens/naive_atlas) default parameters (31). This workflow combines the highly automated ATLAS workflow (https://github.com/metagenome-atlas/atlas; designed only for prokaryotes) (44) with the taxonomically agnostic (but less automated) VEBA v2 (https://github.com/jolespin/veba) workflow (45). Genes were annotated for each MAG using GhostKOALA (v 3.1) (46). All downstream analyses were performed using Python v3.9.7 with the base packages, pandas (v2.2.3), and numpy (v1.26.4). Plots were generated using matplotlib (v3.6.2) and seaborn (v0.12.1). Statistical tests were performed using SciPy (v1.13.1) unless specified otherwise.

### Ecological diversity and interactions

The pooling of DNA replicates before sequencing resulted in a single replicate per time point for metagenomic analyses; thus, to enable statistical analysis, time points were grouped into “phases” using alpha diversity (Shannon diversity and Simpson evenness index; scikit-bio v0.6.3 using function alpha) metrics. These phases were as follows: week 1 (*n* = 4, TP0–TP3, hereinafter early), week 2 (*n* = 4, TP4–TP7, hereinafter middle), and weeks 3 and 4 (*n* = 2, TP8–TP9, hereinafter late) (Fig. 1A). Within each phase, the metagenomic data from each time point were used as pseudo-replicates for downstream analysis. A Shapiro-Wilk test was utilized to assess the normality of the data. Because the data were normally distributed, a one-way analysis of variance (ANOVA) test was performed to assess the overall alpha diversity shifts between phases. A Tukey-HSD test was subsequently performed for assessing pairwise shifts. Beta diversity between the time points was assessed using Aitchison distance, by first performing a centered log ratio transformation and subsequently generating a dissimilarity matrix (scipy.spatial.distance: pdist, squareform, and skbio.stats.composition: clr) (47). To further assess the time point clustering profile, we used the same dissimilarity matrix in a non-metric multidimensional scaling (NMDS) analysis using sklearn.manifold MDS (scikit-learn v1.6.1). We also performed a PERMANOVA analysis (999 permutations) and PERMDISP to assess the effect of variation between time points.

Relative abundances were calculated twice for each sample, once using just eukaryotes and prokaryotes (hereinafter microbiome), and once using just viruses (hereinafter virome). This was performed to prevent large compositional shifts in one MAG type from affecting the apparent relative abundance of MAGs within the other. The transcripts per million (TPM) metric was used for the relative abundance analysis within each sample; because DNA reads were utilized for this analysis, this metric is more accurately described as genomes per million (GPM). Microbiome shifts were evaluated at the order level, whereas virome shifts were assessed at the class level (based on the Baltimore classification).

### Meta-transcriptome mapping

Ribo-minus reads were obtained from the sequencing facility, wherein one sample was removed from subsequent analyses due to extremely low read counts (SMD-RNA-28). Reads were cleaned to remove low-quality regions and adapters using fastp (v0.23.2) (48) using the flags: --qualified_quality_phred 20, --unqualified_percent_limit 10, --length_required 75, --detect_adapter_for_pe --cut_right --cut_right_window_size 5, --cut_right_mean_quality 20. Read quality was verified using fastqc (v0.12.1) (49) both before and after cleaning. Salmon (v0.13.1) was used to build an index (--index) of all genes in the metagenome (binned and unbinned) and to quasi-map the quality-controlled reads against the built index (--validateMappings) (50).

### Differential gene expression

The count matrix obtained from salmon-quasi mapping was subsequently filtered to remove genes with low counts, that is, zero values in more than four samples. We performed differential gene expression analyses using PyDEseq2 (v0.4.8) (51). Differentially abundant genes were identified using TP0 as the control and each of the subsequent time points as the treatment. Normalization factors were calculated (fit_size_function()), followed by genewise dispersions (fit_genewise_dispersion()), fitting dispersion trend coefficients (fit_dispersion_trend()), and finally, the log fold change for each comparison was calculated (fit_LFC). A gene was considered differentially expressed if it had a |fold-change| (hereinafter FC) > 2.0 and an adj-*P* value < 0.05. Wald statistic metrics were saved for use in gene set enrichment analysis (GSEA) (see below). MAGs for downstream analysis were selected based on relative abundances and number of upregulated genes. If a MAG had more than 200 upregulated genes and had a relative abundance >0.10 at any one time point, the MAG was considered a degradation-associated MAG (DAM).

### Gene set enrichment analysis

Gene set enrichment analysis (GSEA) was performed on the four identified MAGs of interest using GSEApy (v1.0.6) (52) and restricted to KEGG pathways and modules to capture coordinated, pathway-level transcriptional shifts. Analyses were done at the KEGG C-Description level. Ranked gene lists for each MAG were generated based on Wald statistics from PyDESeq2. GSEApy was run with the following parameters: --minsize 10, --maxsize 5000, --permutation_num 1000, --seed 42. A gene pathway was considered enriched if it had a normalized enrichment score (NES) > 1.0 and an FDR *q* value < 0.25, which are thresholds commonly used for exploratory GSEA (53). To account for transcriptomic variation across MAGs at different time points, results were grouped according to the early, middle, and late phases used for the metagenomic analysis. A pathway was deemed enriched within a phase if enrichment was observed in at least one of the time points in that phase.

### Arsenic detoxification

Given the high arsenic content of *Sargassum*, we investigated genes involved in its detoxification in the associated microbiome. To initially identify these genes, we performed a DIAMOND (v 2.1.13) blastp (--evalue E-10, --max-target-seqs 0) (54) search against the BacMet database (v2.0) (55). Additionally, to verify the function of each gene with hits to the BacMet database, a phylogenetic approach was performed, with each gene of interest compared against the nr database using DIAMOND blastp (--evalue E-10, --max-target-seqs 0). The hits returned by this analysis were too voluminous for downstream analysis, making the analysis phylogenetically intractable or too large to visualize; therefore, these hits were downsampled. For each query protein, hits to the nr database were downsampled, retaining only the top hit for each taxonomic class (i.e., for each taxonomic class in the resulting hits, just the top hit with the lowest *e* value was retained). For each query, the protein sequences of the hits that remained after downsampling were combined, along with the query protein itself, into a file for downstream analysis. For each of the resulting protein files, MAFFT (v7.520; --auto) (56) was used to generate an alignment and trimAl (v1.5.rev0; --automated1) (57) was used to trim poor quality sequences and positions. Maximum likelihood (ML) phylogenetic trees were generated for each trimmed alignment using iqtree (v2.3.6; -m TEST -bb 1000) (58). Phylogenetic trees were manually assessed to verify the phylogenies. Finally, we assessed the pyDEseq2 results for each of the confirmed arsenic gene annotations.

### CAZyme encoding genes and substrate prediction

For all MAGs in the microbiome, we predicted CAZyme encoding genes, CAZyme gene clusters (CGC), and subsequent substrates using dbCAN (v4.1.4) (59). dbCAN was run on --easy_substrate (-gff_type prodigal, --mode protein, --e_value_threshold 1e-10). Annotated CAZymes were subsequently evaluated for transcriptomic activity based on the pyDEseq2 results. A central focus of this study was to assess both the presence and expression of CAZyme encoding genes involved in the degradation of key *Sargassum*-associated polysaccharides, including fucoidan, alginate, cellulose, and laminarin. In addition, CGCs were examined to infer the polysaccharide substrates they were predicted to target.

## RESULTS

### Sequencing results and metagenome-assembled genome (MAG) statistics

The summary of reads generated in this study is presented in Text S1. A total of 240 MAGs were constructed across all time points. Of this, 41 MAGs were prokaryotic (pMAGs), with 17 classified as “Complete” (completeness > 90% and contamination < 5%, as assessed by CheckM), 19 were classified as “High-quality” (completeness > 70% and contamination < 10%), and 5 were classified as “Medium-quality” (completeness > 50% and contamination < 10%) (Table S1A). A total of 113 were viral (vMAGs), all of which had a geNOMAD viral score > 0.85 and fdr < 0.05 (Table S1B). Two were eukaryotic (eMAGs), both in the fungal genus *Penicillium* and were relatively complete according to BUSCO (eukaryote_odb10) analysis. eMAG01 had a completeness of 80% and eMAG02 a completeness of 94.1 (Table S1C). Finally, 84 were plasmid MAGs (plMAGs), of which 16 contained over 50 protein-coding genes (Table S1D). Previous studies have described the potential of mega-plasmids, which can encode hundreds of CAZyme genes involved in degrading complex polysaccharides (40). However, upon assessing the plMAGs generated in this study, only a small number of CAZymes were identified, unlike the previously identified mega-plasmid (40). Therefore, because the plMAGs did not encode CAZyme genes of interest, they were excluded from downstream analysis.

### Ecological diversity: microbiome and virome dynamics

Alpha diversity metrics were calculated for the microbiome, using Shannon (evenness) and Simpson indices (diversity) (Fig. 1B). Using a Shapiro-Wilk test, the data were observed to be normally distributed. A one-way analysis of variance (ANOVA) was performed on both metrics, and significant shifts were observed (Shannon: *F*-statistic = 5.523, *P* value = 0.0364; Simpson: *F*-statistic = 6.750, *P* value = 0.0236). A Tukey-HSD post hoc analyses showed significant decline in alpha diversity metrics in the late phase, compared to both early (Shannon: adj-*P* value = 0.0415, Simpson: adj-*P* value = 0.0344) and middle phase (Shannon: adj-*P* value = 0.0344, Simpson: adj-*P* value = 0.0256). These results strongly suggest the homogenization of the microbiome degrading *Sargassum* over time. PERMANOVA was used to evaluate the significance of microbial beta-diversity (Fig. 1C) across the three phases, that is, early, middle, and late (pseudo-*F* = 1.98, *n* = 10, *P* value = 0.022, permutations = 999). To confirm these findings, PERMDISP was also calculated (*F* value = 0.32, *n* = 10, *P* value = 0.734, permutations = 999). These results indicate that the shifts in the microbiome are driven by phases and not variation between the samples. Based on the alpha diversity metrics (Fig. 1B), TP3 appears to be an outlier. The TP3 pattern suggests tube-to-tube variation in degradation rate, with its removal from the beta-diversity analysis resulting in increased overlap between the early and middle phases (Fig. S1). However, no technical artifacts (e.g., reduced read count or mapping rates) were detected for TP3, supporting the idea that it is a biologically meaningful early sample with a community composition more characteristic of the late phase. This result highlights the heterogeneity of microbial communities involved in *Sargassum* degradation. Therefore, given the lack of clear evidence for technical artifacts that would necessitate its removal, TP3 was retained in the downstream analysis.

Utilizing GPM values (genomes per million) relative abundances were calculated at the family level for the microbiome, that is, eMAG and pMAG (Fig. 1D; Table S2). Across all phases, members of the families Psychromonadaceae, Marinomonadaceae, and Vibrionaceae consistently dominated the microbial community, though their relative abundances varied markedly by phase (see Text S1 for details, Fig. 1D). Furthermore, we observed a clear decline in the Nostocaceae (cyanobacteria) relative abundance. Viral relative abundances were calculated separately. Notably at TP0, Megaviricetes was the most abundant class at TP0 (~42%), though a marked decrease was observed in later time points. We observed an increase in *Caudoviricetes* abundance, that is, bacteriophages at TP2, and remained the dominant class until the end of the experiment (see Text S1 for details; Fig. 1D; Table S2).

### Differential gene expression analyses

For sequencing details and generated reads see Text S1. A total of 39 RNA-seq samples (four replicates per time point, with one dropout) were mapped to genes from the Naïve ATLAS constructed MAGs and unbinned contigs derived from each sample (totaling 606,154 genes). After filtering for low abundance, 45,231 genes remained for downstream analysis. Upon examining gene abundance across each sample, we observed considerable variability between replicates within individual time points, likely attributed to each sample being derived from independent tubes. To mitigate the impact of this variation, a more stringent threshold for differential expression was applied (|fold change| > 2.0, adj-*P* value < 0.05). Furthermore, to enable integration between the metatranscriptomic and metagenomic data sets, all downstream analysis was conducted according to the phases identified using the metagenomic samples. The results from each of the metatranscriptomic time points were summarized into the phases observed in the metagenome: that is, if a DEG was observed at a single time point, then it was considered significantly expressed at the phase level, even if it was not differentially expressed in any of the other time points in that phase. Overall, differential gene expression analysis was performed at each time point using TP0 as the reference. In total, 14,897 genes were identified as differentially expressed genes (DEGs) (|fold change| > 2.0, adj-*P* value < 0.05) across any of the time points (Table S3). Of these, 7,882 DEGs originated from MAGs, and 7,003 were from unbinned contigs, the latter of which were excluded from downstream analysis. Of the MAG-derived DEGs, 4,760 were upregulated and 3,475 were downregulated.

### Identifying active members of the microbiome

**Fig 2:**
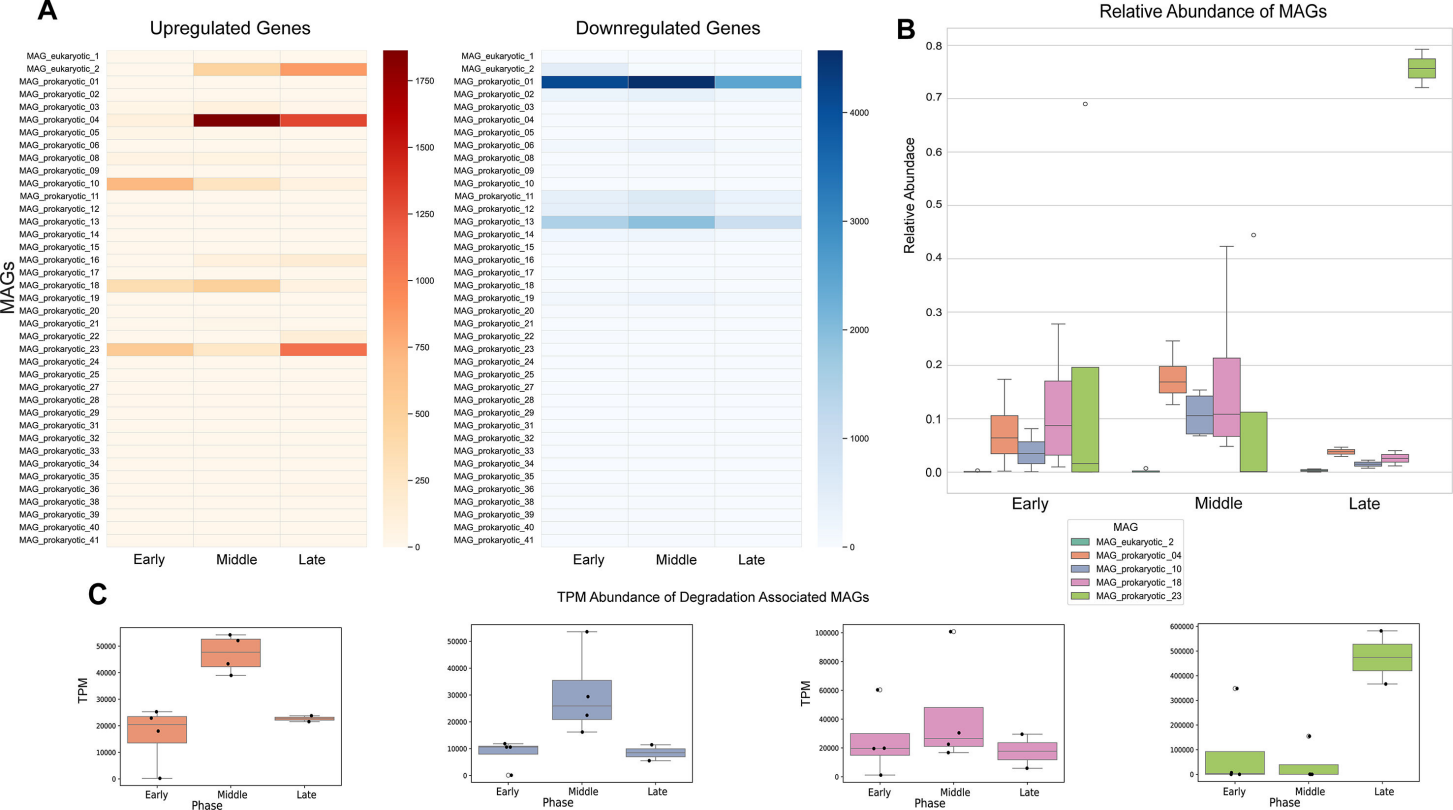
Selection of DAMs. (**A**) Heatmap showing the number of upregulated (orange) and downregulated (blue) genes in microbiome-associated MAGs in each phase compared to TP0. The *y*-axis shows the MAGs and *x*-axis shows the phase, and the hues show the number of upregulated or downregulated genes. A MAG is of interest if it has at least 200 upregulated genes. Five MAGs passed this initial filter and are presented in panel B. (**B**) Relative abundances (microbiome, same values as Fig. 1D) presented as a box plot for each of the MAGs of interest. The *y*-axis shows the relative abundance of each MAG, and the *x*-axis shows the phase. The colors have been presented in the legend. MAGs were considered DAMs if in at least one phase they had a relative abundance greater than 0.1. (**C**) GPM based abundance of the four DAMs, with the same color scheme as image (**B**). GPM values have been presented on the *y*-axis and the *x*-axis represents phase.

By integrating the metagenomics and metatranscriptomics data, we identified four prokaryotic MAGs that were the most active during the degradation experiment. A MAG was classified as a degradation-associated MAG (DAM) if it had at least 200 upregulated genes in the metatranscriptomic data during any phase (Fig. 2A) and exhibited a relative abundance greater than 0.10 during any phase (excluding early phase) in the metagenomic data (Fig. 2B). Four DAMs passed these two thresholds, all of which were pMAGs (pMAG04, pMAG10, pMAG18, and pMAG23). pMAG04 (middle phase: 0.168 ± 0.050; 1,324 upregulated genes) and pMAG10 (middle phase: 0.105 ± 0.044; 752 upregulated genes) were both annotated at the genus level as *Psychromonas*. pMAG18 (middle phase: 0.108 ± 0.172; 516 upregulated genes) was annotated at the genus level as *Marinomonadaceae*. Finally, pMAG23 (late phase: 0.756 ± 0.050; 633 upregulated genes) was annotated at the species level as *Vibrio nitrifigilis*. A significant decline was observed in the relative abundance of pMAG04**,** pMAG10, and pMAG18 during the late phase. However, GPM-based assessment indicated that the absolute abundance of these pMAGs likely remained relatively stable (Fig. 2C). The apparent reduction in relative abundance in the late phase is therefore driven primarily by the marked increase in pMAG23 abundance.

### Pathway level enrichment analysis in degradation-associated MAGs

**Fig 3:**
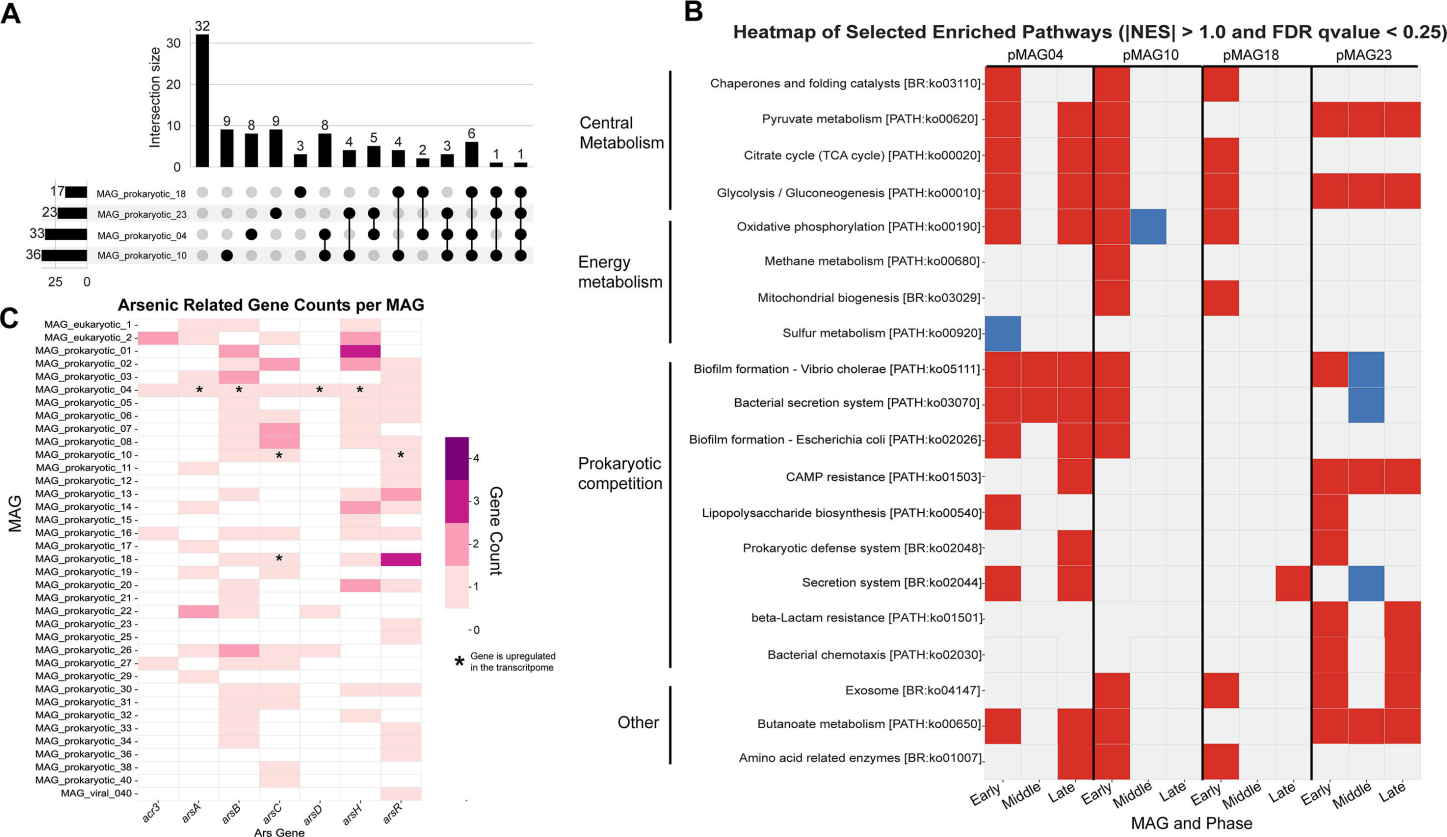
Enriched gene pathways in DAMs. (**A**) UpSet plot showing intersections of enriched metabolic pathways identified in the DAMs. Each set (horizontal bars, left) represents the total number of significantly enriched pathways detected in an individual MAG. Vertical bars (top) represent the size of intersections, that is, the number of pathways shared between specific combinations of MAGs, with filled dots indicating which MAG(s) are included in the intersection. (**B**) A heatmap showing a subset of key pathways enriched in DAMs (see Fig. S1 for all pathways). The *y*-axis shows the pathway (based on C-level description KEGG), further grouped into general categories. The *x*-axis shows the pMAG and the respective phase. Red implies that the pathway is upregulated in the phase, blue implies pathway is downregulated in the phase, and gray indicates no significant change. (**C**) A heatmap showing the distribution of arsenic detoxification genes in all generated MAGs of interest (*y*-axis). Gene names are on the *x*-axis and the legend details the colors used, which is based on the copies of each gene in a MAG. Genes marked with an asterisk represent genes that are upregulated in the transcriptome.

A total of 95 KEGG pathways (C-level description) met the adequate criteria for gene set enrichment analysis: that is, minimum gene requirement (*n* = 20). GSEA was conducted independently for each pMAG to identify pathways enriched at different time points. These results were then consolidated into broader phase-based categories to align with the metagenomic analyses. A pathway was considered enriched if the normalized enrichment score (NES) was greater than 1.0 and the FDR *q* value was below 0.25. Pathways were considered downregulated if NES was less than −1.0 with the same FDR threshold (Table S4). Seventeen pathways were enriched in at least one phase in pMAG18, 23 in pMAG23, 33 in pMAG04, and 36 in pMAG10 (Fig. 3A). Although several pathways were enriched across multiple DAMs, the phase in which these pathways were enriched varied (Fig. S2). We quantified the number of upregulated and downregulated pathways for each pMAG in the early, middle, and late phases. All four DAMs were active in the early phase; however, only two (pMAG04 and pMAG23) were also active in the late phase, although their activity dipped in the middle phase.

### DAM enrichment for prokaryotic competition and other notable pathways

We observed nine prokaryotic competition pathways that were enriched in at least one DAM (Fig. 3B). Notably, biofilm formation*—Vibrio cholerae* (ko05111) was upregulated in all three phases for pMAG04, early phase for pMAG10, and early phase for pMAG23; however, it was downregulated in the middle phase. Biofilm formation*—Escherichia coli* (ko02026) was upregulated in the early and late phases for pMAG04, and in the early phase for pMAG10. The bacterial secretion system pathway (ko0370) was enriched in all three phases for pMAG04, and in the early phase for pMAG10. Whereas this pathway was downregulated in the middle phase for pMAG23, another secretion system pathway (ko02044) was enriched in the early and late phases for pMAG04, and in the late phase for pMAG18. Finally, lipopolysaccharide (LPS) biosynthesis was upregulated in the early phase for both pMAG04 and pMAG23. Likewise, we observed enrichment in pathways associated with defensive and resistance pathways. For example, the cationic antimicrobial peptide (CAMP) resistance pathway (ko01503) was enriched in the late phase for pMAG04 and was enriched at all three phases for pMAG23. Prokaryotic defense pathway (ko02048) was enriched in the late phase for pMAG04, and in the early phase for pMAG23. Beta-lactam resistance pathway (ko01501) and bacterial chemotaxis (ko02030) were upregulated in the early and late phases for pMAG23. Finally, we observed that the exosome pathway (ko04147) was upregulated in the early phase for pMAG10, pMAG18, and pMAG23, but was upregulated in the late phase for pMAG23.

We also assessed sulfur, methane, and nitrogen metabolism that have been implicated in the *Sargassum-*microbiome symbiosis and degradation dynamics (15). We observed that in pMAG04, there was a downregulation in the sulfur metabolism pathway in the early phase; however, no other DAM showed any significant changes. For methane metabolism, we observed upregulation in the early phase for just pMAG10. Finally, no shifts were observed in nitrogen metabolism in any of the four pMAGs. Furthermore, none of the *nif* genes showed differential expression in the nitrogen-fixing pMAG23.

### Arsenic detoxification genes

The sequestration of arsenic by *Sargassum* presents a unique challenge for associated microbes that utilize this seaweed as a substrate. Among the 43 MAGs (microbiome) analyzed, 36 contained at least one arsenic detoxification gene, including all four DAMs (Table S5). In the four DAMs, phylogenetics was used to verify the annotation of these genes (Supplemental file). Only pMAG04 contained the complete *arsRABCD* operon, with *acr3* and *arsH* also identified (Fig. 3C). pMAG18 encoded six of these genes but lacked *acr3*, *arsA*, and *arsD*. pMAG10 had only *arsB*, *arsC*, and *arsR*, whereas pMAG23 contained just a single copy of *arsR*.

We utilized the differential gene expression results to assess shifts in arsenic detoxification genes across the three phases. Only three pMAGs showed upregulation of arsenic genes, all of which were DAMs (pMAG04, pMAG10, and pMAG18 [asterisks in Fig. 3C]). In pMAG04, four arsenic genes were upregulated (*arsA*, *arsD*, *arsH*, and *arsB*), all in the middle and late phases. In contrast, in pMAG18, *arsC* was upregulated at early and middle phases but not at late phases, and *arsR* was also upregulated at early and middle phases. Similarly, in pMAG10, only *arsC* was upregulated in the early phase. Notably, pMAG23 encoded only one *arsR* gene, but its differential expression was not found.

### CAZymes involved in the degradation of *Sargassum* polysaccharides

dbCAN was used to predict CAZymes, and CAZyme-associated genes such as sulfatases, transcription factors, and transporters (TCs). In this study, we focused on CAZymes involved in degrading recalcitrant polysaccharides in *Sargassum*, that is, fucoidan, alginate, cellulose, and laminarin. In this work, any gene predicted as a CAZyme by at least one of the three homology search tools used by dbCAN was included in downstream analyses. Overall, 29,718 CAZymes were predicted in the metagenome (Table S6A). dbCAN predicted 1,324 CAZyme gene clusters (CGCs); however, only 185 CGCs had an annotation in the PUL database (~14%) (Fig. S3).

**Fig 4:**
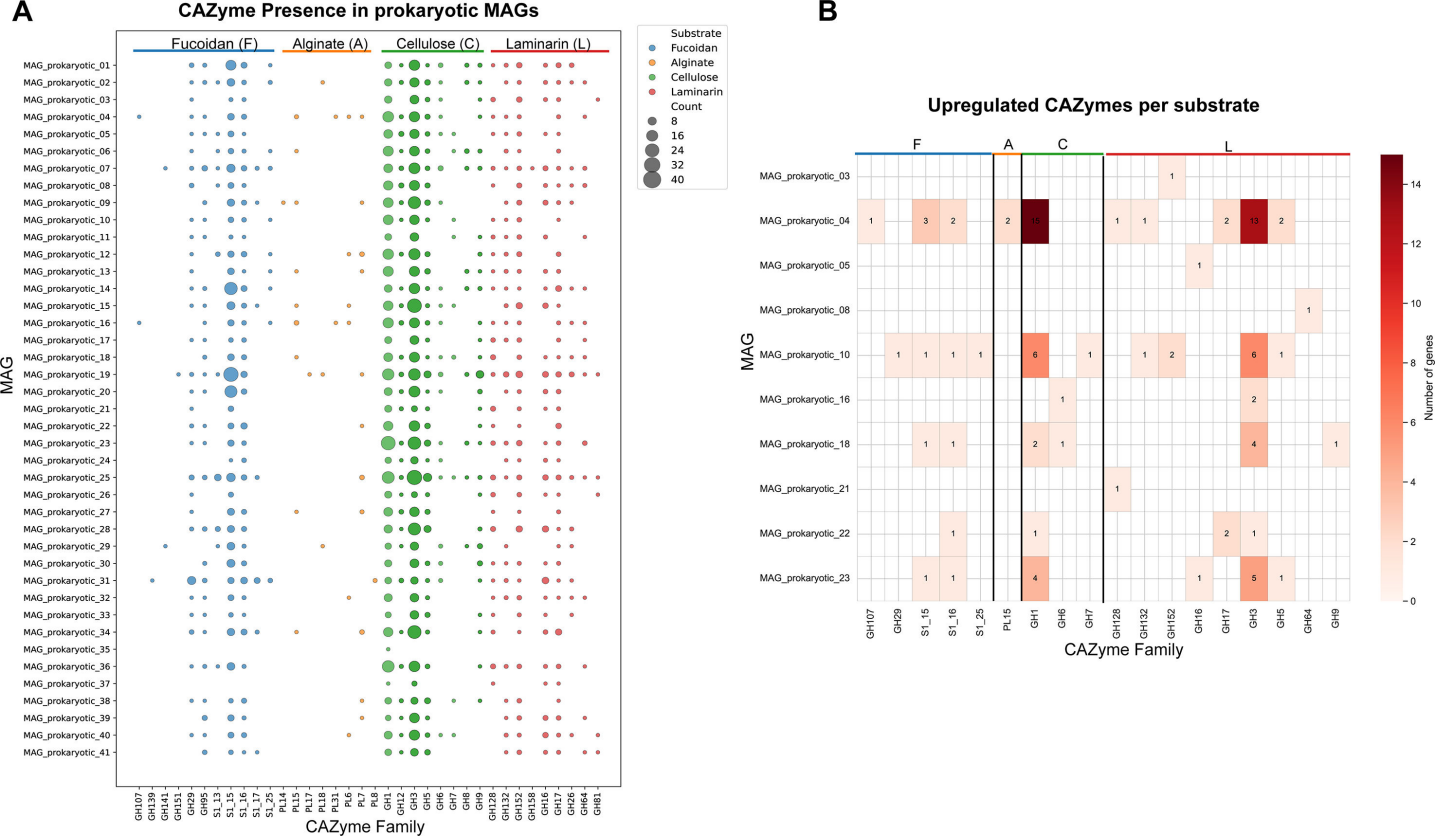
CAZyme distribution in prokaryotes. (**A**) Dotplot showing the distribution of fucoidan (blue), alginate (orange), cellulose (green), and laminarin (red) associated genes. The gene family has been shown on the *x*-axis and the MAG is presented on the *y*-axis. A legend showing the CAZyme type and count has been shown on the right. (**B**) Heatmap showing upregulated CAZymes involved in degrading substrates of interest. MAGs with upregulated genes are presented on the *y*-axis and the CAZyme family is presented on the *x*-axis. At the top of the graph, CAZymes have been grouped by substrate.

A total of 113 fucoidan-related GH genes were identified, primarily belonging to GH29 (56 genes) and GH95 (51 genes) (Fig. 4A). These genes were widely distributed across the MAGs; notably, pMAG31 (Bacteroidaceae) contained the highest number of fucoidan-related GH genes (17 genes), including 13 GH29, 3 GH95, and 1 GH139. A total of 310 sulfatases from families associated with fucoidan degradation were also identified. Like the fucoidan-related GH genes, sulfatases were broadly distributed, with pMAG19 containing the greatest number (50 sulfatases, comprising 42 S1_15, 7 S1_16, and 1 S1_13 gene). In contrast, only 160 alginate lyases (PL family) were identified across the microbial MAGs, with just 20 of the 41 pMAGs encoding alginate lyase genes. Furthermore, we analyzed the 528 cellulose enzymes and 360 laminarin-degrading enzymes identified. Some enzyme families, namely GH3, GH5, GH8, GH9, and GH12, have been associated in both laminarin and cellulose degradation. These enzymes were widely distributed in the microbiome (Fig. 4A).

The metatranscriptomic data were used to assess the activity of these enzymes across each of the MAGs (Fig. 4B; Table S6B). Overall, we found that 98 CAZymes were upregulated, out of 1,471 CAZymes identified as acting on our substrates of interest. Of the fucoidan-degrading CAZymes, 14 were upregulated. Within the GH family, only four genes were upregulated, all in DAMs. In pMAG04, GH107 and GH95 genes were upregulated during the middle and late phases, while in pMAG10, one GH95 gene (across all three phases) and one GH25 gene (early and middle phases) were upregulated. Of the detected sulfatases, 12 were upregulated, 11 of which were found in the DAMs. In pMAG04, three S1_15 and two S1_16 genes were upregulated; in pMAG10, one S1_25, one S1_15, and one S1_16 gene were upregulated; in pMAG18, one S1_15 gene was upregulated; and in pMAG23, one S1_15 and one S1_16 gene were upregulated. The only non-DAM that had an upregulated sulfatase gene (S1_16) was pMAG22. Only two alginate lyase genes, both belonging to the PL15 family, were upregulated in the DAM pMAG04.

A total of 50 CAZymes associated with laminarin degradation were identified in this study. Notably, 31 GH3 family enzymes (also potentially involved in cellulose degradation), which show exo-ß-glucosidase activity, were upregulated, of which 26 were in DAMs. In pMAG23 and pMAG04, four and eleven enzymes (respectively) were upregulated across the duration of the experiment and were notably upregulated at the late phase. Whereas in pMAG18 and pMAG10, four and six enzymes were upregulated during the early phase. We also identified GH128, GH132, GH152, GH16, GH17, and GH5 enzymes upregulated in the MAGs (Fig. 4B; Table S6B).

Thirty-one upregulated CAZymes associated with cellulose degradation were identified. Twenty-eight of these CAZymes belonged to the GH1 family, which are enriched in enzymes like ß-glucosidase that act on cellodextrins or soluble ß-glucosides. Fifteen enzymes were upregulated in pMAG04, primarily in the middle and late phases. In pMAG10, six enzymes were upregulated, primarily in the early phase. Similarly, in pMAG18, two enzymes were also upregulated in the early and middle phases. Finally, in pMAG23, four enzymes were upregulated only in the late phase.

Finally, we assessed mannitol catalyzing genes, mannitol 1-phosphatase dehydrogenase (*mtlD*), a mannitol specific phosphotransferase gene (*mtlA*), and the mannitol operon repressor (*mtlR*) in our DAMs. Notably, both pMAG04 and pMAG23 had upregulated genes in the operon and enriched glycolysis and gluconeogenesis pathway. Notably, in pMAG04, three copies of the *mtlA* transporter were upregulated at the middle and late phases, whereas one copy was only upregulated at late. The *mtlR* operon repressor was upregulated in the middle and late phases. However, in pMAG23, one copy of *mtlA* and one copy of *mtlD* were upregulated in all three phases. Furthermore, preliminary compositional analysis showed a significant decline in mannitol concentration during the experiment period; however, variation between replicates made it challenging to draw robust biological insights from these data (for details, see Text S2).

## DISCUSSION

### Shifts in the microbiome drive homogenization

We generated MAGs from prokaryotes (pMAGs), eukaryotes (eMAGs), viruses (vMAGs; the virome), and plasmids (plMAGs) from the *S. filipendula* microbiome during the degradation period. The lack of biological replicates in the metagenomic data led us to (successfully) evaluate community shifts using a phase-based approach: TP0–TP3 (early phase), TP4–TP7 (middle phase), and TP8–TP9 (late phase). The degradation experiment lasted 28 days, with each phase spanning approximately 1 week. Although we used the benthic *S. filipendula* in our experiment, past work demonstrates that this species and pelagic *Sargassum* share a similar polysaccharide composition (42, 43).

Consistent with previous studies, we observed a diverse microbiome during the early and middle phases of this experiment, followed by a marked decline in diversity and evenness in the late phase when the community was dominated by a small number of MAGs (15, 23, 27). Unlike earlier studies where these shifts occurred within 48 h (23), this shift emerged only in the late phase. In fresh *S. filipendula* (TP0), cyanobacterial MAGs were abundant but declined rapidly during the early phase. In the following phases, the microbiome was dominated by four pMAGs, two *Psychromonas*, one *Marinomonas*, and one *Vibrio nitrifigilis* MAG (Fig. 2B). Notably, in the late phase, *V*. *nitrifigilis*, a nitrogen-fixing species (60), became the clear dominant taxon, comprising ~80% of the community. *Vibrio* dominance in stranded *Sargassum* has been documented previously, which may be attributed to its ability to utilize diverse carbon sources and its opportunistic nature (24, 61). However, although an expansion in *V. nitrifigilis* abundance was observed, other DAMs appeared to persist at the late phase (Fig. 2C).

The four DAMs (all pMAGs) exhibited the highest number of upregulated genes compared to TP0 (Fig. 2A), accompanied by enrichment of central metabolic pathways including glycolysis, gluconeogenesis, the TCA cycle, and amino acid metabolism (Fig. 3B; Fig. S1). Transcriptomic profiles also suggested functional divergence among these MAGs. For example, pMAG10 (*Psychromonas*) and pMAG18 (*Marinomonas*) were highly active during the early phase, with 30 and 18 pathways upregulated, respectively, but exhibited limited activity in the middle and late phases, indicating early degradation roles followed by competitive exclusion. In contrast, pMAG04 (*Psychromonas*) and pMAG23 (*Vibrio nitrifigilis*) maintained activity throughout the experiment, showing comparable pathway enrichment through early to late phases.

### Divergent competitive strategies and viral influence drive microbiome homogenization in degrading *Sargassum*

Microbial composition in degrading *Sargassum* is shaped by both environmental conditions and microbial interactions. Upon stranding of the pelagic species, macroalgal tissues are exposed to variable temperatures, oxygen depletion, and nutrient shifts, factors that create selective pressures and influence microbial succession (62). Concurrently, the breakdown of algal polysaccharides and the release of dissolved organic matter provide diverse substrates that can either promote cooperation or drive competition among microbes (62). To disentangle environmental effects from biological interactions, our study attempted to minimize environmental variability. In this study, we utilized the benthic *Sargassum filipendula* as a model, owing to its availability, broad range (Caribbean to the Northeast USA), and its similar polysaccharide composition to pelagic *Sargassum* (35, 42, 43) to assess microbiome dynamics in a closed *in vitro* environment. Our findings suggest that the microbial community is not primarily structured by syntrophic cooperation, as initially hypothesized, but rather by competitive dynamics. Although some microbial associations were observed, the dominance of only four pMAGs points to a selective, competitive process.

We assessed the role of direct prokaryotic competition in driving microbiome homogenization during *Sargassum* degradation. Among the DAMs, nine pathways associated with competition were enriched, including biofilm formation, bacterial secretion systems, lipopolysaccharide synthesis, CAMP resistance, and general prokaryotic defense (63, 64). Over the course of the experiment, we observed divergent strategies among the DAMs. pMAG18 exhibited minimal enrichment, with only the secretion system pathway enriched during the late phase. In contrast, pMAG10 displayed strong early-phase enrichment of competition-related pathways, particularly biofilm formation and secretion systems, but showed no enrichment in later phases and lacked expression of resistance or defense mechanisms. Although pMAG23 appeared to be the dominant MAG within the system, primarily in the late phase, it exhibited a mixed competitive strategy: biofilm formation was enriched in the early phase but downregulated in the middle phase, with no significant changes in the late phase. However, defensive pathways were consistently upregulated in pMAG23, CAMP resistance across all phases, beta-lactam resistance in the early and late phases, and the prokaryotic defense pathway in the early phase. In contrast, pMAG04 followed a more aggressive strategy, showing consistent enrichment of biofilm formation and secretion system pathways, with resistance mechanisms such as CAMP resistance and prokaryotic defense becoming enriched only in the late phase. These patterns suggest that pMAGs use diverse strategies for competition, such as pMAG04, that employs active interference strategies to compete, whereas others such as pMAG23 may adopt a more defensive approach (65).

In addition to prokaryotic interactions, our results suggest a potential role for viruses in shaping microbial community dynamics. The fresh *Sargassum* (TP0) virome was dominated by vMAGs in class *Megaviricetes*, including members annotated as *Algavirales* and *Imitevirales*, which are known to infect algae (66). These viruses showed a rapid decline early in the experiment (Fig. 1D), suggesting they may have infected the *Sargassum* host and declined following host death. From TP2 onwards (still in the early phase), the samples were dominated by *Caudoviricetes*, an abundant class of bacteriophages (67). Although specific phage-host relationships were not characterized in this study, previous work has highlighted the role of phages in modulating microbial community structure (22).

### Substrates within *Sargassum* that are utilized by the microbiome

Alginate is a linear co-polymer comprised of two monosaccharide sugar acids, β-d-mannuronic acid (M) and α-l-guluronic acid (G), assembled in consecutive mannuronic units (M-blocks), guluronic units (G-blocks), or alternating units (MG-blocks) depending on multiple factors including seaweed species, tissue type, and location/growth conditions (68). In the meta-transcriptomic data, the only upregulated PL genes were in pMAG04. From this information, we suggest that pMAG04 (*Psychromonas* sp.) actively degraded alginate in our seaweed substrate. Fucoidan from *Sargassum* species is a recalcitrant and heavily sulfated polysaccharide, comprised of numerous monosaccharides such as fucose, glucose, and galactose (69). Whereas prior work identified fucoidan-degrading CAZymes encoded on a *Lentimonas* mega-plasmid (Verrucomicrobiota) (40), these genes did not degrade fucoidan completely. Regardless, neither Verrucomicrobiota nor large CAZyme-carrying plasmids were detected in this study. However, generally, fucoidan-degrading enzymes appeared to be ubiquitous in the microbiome. In the transcriptomic data, only 4 fucoidan-related CAZymes and 12 sulfatases were upregulated, all in pMAG04 and pMAG10, suggesting potential fucoidan degradation capabilities in our system. This study further highlights that gene presence does not necessarily imply expression, underscoring the limitations of inferring microbial activity based only on metagenomic data. For instance, Bacteroidaceae (pMAG31), which are known to be polysaccharide degraders (40, 70), harbored numerous fucoidan-related CAZymes; however, none of these genes showed differential transcriptomic activity.

Furthermore, we observe a prominent number of GH1 (cellulose) and GH3 (laminarin and cellulose) enzymes, both exo-ß-glucosidases, upregulated in the DAMs. However, the majority of these were identified in pMAG04, with pMAG04 and pMAG23 showing upregulation of these genes in the middle and late stages of the study. Laminarin, a storage molecule in brown-algal seaweeds, contains chains that terminate with mannitol and those that terminate with glucose (71); we observe transcriptomic evidence of mannitol catabolism. Mannitol-catalyzing genes (*mtlA*, *mtlD*, and *mtlR*) are required for an organism to integrate mannitol into glycolysis and gluconeogenesis pathways (72, 73). Both pMAG23 and pMAG04 had upregulated genes in mannitol catabolism, although pMAG04 showed this trend only in the middle and late phases, whereas pMAG23 showed enrichment in all three phases, suggesting active mannitol catabolism. We can therefore speculate that these bacteria may be degrading laminarin, releasing mannitol, which is subsequently utilized for its metabolism.

### Host-sequestered arsenic may also drive and limit substrate degradation

In addition to the foul odors associated with hydrogen sulfide (H_2_S) production during *Sargassum* degradation, the ability of this seaweed to sequester toxic metals such as arsenic (As) poses serious environmental and public health risks (74). Rafts of *Sargassum* accumulate significant quantities of arsenic (62.2 mg/kg, but in some cases up to 142 mg/kg) (75), which may leach into surrounding soil and coastal ecosystems during degradation. Given this observation, we hypothesized that microbes involved in *Sargassum* degradation would encode genes associated with arsenic detoxification (76). Indeed, our metagenomic analysis revealed that most MAGs within the microbiome harbored at least one arsenic detoxification or efflux gene, although only pMAG04 contained the full complement of the *arsRABCD* operon (77) (Fig. 3C). Notably, the most dominant MAG, pMAG23, possessed only *arsR*, a transcriptional regulator of the arsenic response.

Arsenic detoxification appeared to be limited to the DAMs. In pMAG10 and pMAG18, we observed upregulation of the *arsC* gene, which encodes arsenate reductase and facilitates the conversion of arsenate [As(V)] to arsenite [As(III)], a more soluble and exportable form (78). This response was confined to the early phase in pMAG10 but spanned the early and middle phases in pMAG18. Furthermore, *arsR* was also upregulated in pMAG18. In contrast, pMAG04 exhibited a broader detoxification response, with upregulation of *arsA*, *arsB*, *arsD*, and *arsH* in the middle and late phases. Whereas *arsA*, *arsB*, and *arsD* contribute to arsenite production and efflux, the expression of *arsH* suggests additional arsenic resistance capability (79). This expression pattern, combined with pMAG04 being the sole active alginate degrader, highlights the challenge of degrading polysaccharides from *Sargassum*. Both alginate and fucoidan have been implicated in sequestering arsenic (80); therefore, we hypothesize that as microbes degrade this polysaccharide, they must detoxify the released arsenic.

### Outcomes of degradation-associated pMAGs (DAMs)

The DAMs exhibited distinct ecological strategies during *Sargassum* degradation. The MAG pMAG04 (*Psychromonas*) emerged as an active degrader, showing sustained transcriptional activity across phases, active alginate degradation, laminarin-associated carbon utilization, and robust arsenic detoxification. The latter likely reflects the presence of arsenic released during alginate breakdown, suggesting a key role in the degradation microbiome. Although pMAG04 and pMAG10 are both in the genus *Psychromonas* and display similar middle-phase dominance based on their abundance in the metagenome data, their transcriptional profiles diverged substantially, indicating functional specialization. Whereas pMAG10 exhibited a strong early-phase transcriptional response followed by limited activity, pMAG04 maintained expression of polysaccharide-degrading, microbial competition, and stress-response pathways through the middle and late phases. The DAMs (pMAG10 and pMAG18) with relatively high abundance in the middle phase but weak transcriptomic activity likely reflect metabolic maintenance or homeostasis rather than active upregulation of core energy pathways, following an initial early-phase transcriptional response to newly available substrates or environmental changes (i.e., degrading *Sargassum*). As degradation progressed into the late phase, the capacity of pMAG04 to utilize alginate may have resulted in the observed upregulation of metabolic genes in this MAG. This MAG became dominant in the late phase likely due to its ability to degrade cellulose, and despite lacking broad arsenic resistance capabilities, suggesting that arsenic stress was not a major driver of microbiome composition at the sampled time points. The other DAMs (pMAG10 and pMAG18) remained largely in a homeostatic state in the late phase.

### Natural *Sargassum* degradation provides key insights and considerations for microbiome and enzyme engineering

The recurring mass *Sargassum* stranding events in the Caribbean have sparked growing interest in harnessing these seaweeds as sustainable and viable feedstocks of the future. In this regard, we present a foundational assessment using the benthic *S. filipendula* as a model system. Consistent with previous observations, we find that as “stranding” (i.e., in our experimental system) and degradation proceed, the *Sargassum* microbiome becomes increasingly homogenized (15, 23). Whereas species composition among different *Sargassum* species and populations may vary, we postulate that members of the genus *Vibrio* will thrive due to their metabolic flexibility (61).

Using a combination of metagenomic and meta-transcriptomic, we identified several key drivers of microbiome dynamics during *Sargassum* degradation. First, we demonstrate that the mere presence of fucoidan-degrading genes does not guarantee breakdown of all complex polysaccharide over the duration of our experiments. For instance, pMAG31, despite encoding the largest number of fucoidan-associated CAZymes, none were differentially expressed, suggesting that the complex structure of fucoidan makes it energetically too expensive to degrade when other simpler polysaccharides are available. In addition, we observe that β-glucan degrading enzymes (e.g., GH1 and GH3) could be relevant to the utilization of glucose from readily accessible cellulose/laminarin and/or release of mannitol from laminarin reducing ends. In a competitive microbial community, taxa capable of degrading simpler polysaccharides will likely outcompete those specialized for more recalcitrant substrates, as observed with pMAG23. We also observe active degradation/metabolism of alginate and mannitol, linking changes in seaweed composition with the likely microbial degraders.

Second, our data reveal signatures of active microbial competition. This is evidenced by the enrichment of pathways involved in biofilm formation, lipopolysaccharide (LPS) synthesis, and other prokaryotic defense mechanisms (65). Third, we cannot rule out the role of viruses in shaping microbial community structure. Shifts in viral populations, particularly the rise of bacteriophages over time, may reflect selective pressure on dominant taxa through phage predation (22). Finally, the presence of toxic metals, particularly arsenic, poses an additional challenge. Both fucoidan and alginate are known to sequester arsenic in *Sargassum* (81), and degradation of these polysaccharides likely exposes microbes to elevated arsenic levels, requiring active detoxification of this metalloid, making these substrates less desirable.

This study provides a preliminary but functionally detailed view of microbial processes during natural *Sargassum* degradation. Whereas the identity of the key microbial players may shift across seasons, geographies, or environmental conditions, we propose that the broader ecological dynamics—that is, competitive substrate use, functional bottlenecks, viral predation, and metal toxicity—are likely to converge on similar outcomes across systems. This prediction can inform the engineering of specialized enzymes to degrade seaweed polysaccharides or the engineering of microbes to degrade *Sargassum*.

### Study caveats and future directions

Despite signatures of microbial degradation in the multi-omics data, *Sargassum* fronds largely retained their structure, indicating that degradation was relatively limited even after 28 days. This result may indicate the need for longer time periods to study the degradation cycle under lab conditions. Furthermore, because samples were taken from individual tubes rather than a larger shared culture, variation was introduced between replicates and time points. Additionally, due to the low DNA yield from each sample, time point replicates were pooled for sequencing. To address these issues and allow statistical analysis, we applied a phase-based consensus approach when interpreting results. For future bioreactor experiments, establishing a system that enables repeated sampling from the same culture would help reduce this variability. However, homogenization of the microbiome remains a potential concern because it may lead to the loss of valuable degraders. This is particularly relevant for complex substrates such as fucoidan, which may be more energetically intensive to degrade or require prior degradation of other matrix components to become bioavailable. Consistent with this idea, previous studies have suggested that complex polysaccharides such as fucoidan are degraded by a polymicrobial consortia. In a closed system where no new microbes are introduced, even organisms with the genomic capacity for fucoidan degradation may fail to persist if substrate accessibility is limited. Therefore, identification of natural *Sargassum* degradation sites becomes more valuable. Complementary culture-based experiments could help isolate microorganisms, both prokaryotic and eukaryotic, capable of breaking down complex polysaccharides.

## Data Availability

All raw sequencing data (DNA and RNA) utilized in this study are available in the NCBI SRA database under BioProject accession number PRJNA1415266.

## SUPPLEMENTAL MATERIAL

Supplemental text 1 aem.02357-25-s0001.docx Supplemental results.

Supplemental text 2 aem.02357-25-s0002.docx Preliminary composition analysis.

Supplemental figures aem.02357-25-s0003.pdf Fig. S1 to S4.

Supplemental legends aem.02357-25-s0004.docx Legends for all supplemental material.

Supplemental file aem.02357-25-s0005.pdf Phylogenetic trees of arsenic detoxification genes in DAMs.

Table S1 aem.02357-25-s0006.xlsx Summary of all prokaryotic MAGs, viral MAGs, eukaryotic MAGs, and plasmid MAGs generated in this study.

Supplemental tables aem.02357-25-s0007.xlsx Tables S2, S3, S5, S7, and S8.

Table S4 aem.02357-25-s0008.xlsx Gene set enrichment analysis results for the four degradation associated MAGs.

Table S6 aem.02357-25-s0009.xlsx CAZymes annotated by dbCAN for each prokaryotic MAG; upregulated CAZymes associated with substrates of interest and phase in which gene is upregulated; dbCAN based CAZyme gene cluster annotation.

ASM does not own the copyrights to Supplemental Material that may be linked to, or accessed through, an article. The authors have granted ASM a non-exclusive, world-wide license to publish the Supplemental Material files. Please contact the corresponding author directly for reuse.
