# Supplementary material for: Rafts of change: microbial and functional dynamics in simulated Sargassum strandings
Source: Appl Environ Microbiol. 2026 Mar 31;92(4):e02357-25. doi: 10.1128/aem.02357-25 (PMC13101531; doi:10.1128/aem.02357-25)
Supplement: Supplemental text 1 — Supplemental results. [file aem.02357-25-s0001.docx]

**Supplemental Text 1: Rafts of Change: Microbial and Functional Dynamics in Simulated *Sargassum* Strandings**

**Results**

*Reads generated for DNA and RNA*

Metagenomic short read sequencing produced 96-166 million read pairs per sample. After de-duplicating, filtering, and cleaning, 59-100 million read pairs remained per sample [**Supplemental Table 7**]. Transcriptomic short read sequencing produced 7-115 million read pairs per sample, with the average being 56 million read pairs.

*Beta diversity*

In addition to the beta-diversity analysis using all timepoints, and due to TP3 being a potential biological outlier, we conducted additional beta-diversity analysis without TP3. PERMANOVA was used to evaluate the significance of the microbial beta-diversity without TP3 [**Supplemental Figure 1**] across the three phases, i.e., early, middle, and late (pseudo-F = 2.13, n =9, *p-value* = 0.028, permutations = 999). To confirm these findings, PERMDISP was also calculated (F-value = 1.18, n =9, *p*-value = 0.289, permutations = 999).

*Microbiome composition*

In the early phase, Psychromonadaceae exhibited the highest mean relative abundance (0.206 ± 0.148), followed by Marinomonadaceae (0.114 ± 0.156) and Nostocaceae (0.101 ± 0.121). During the middle phase, Psychromonadaceae again had the highest relative abundance (0.357 ± 0.093), followed by Marinomonadaceae (0.169 ± 0.187) and Vibrionaceae (0.089 ± 0.204). In contrast, the late phase was characterized by a sharp increase in Vibrionaceae, which dominated with a mean relative abundance of 0.842 ± 0.023, while Psychromonadaceae (0.071 ± 0.022) and Marinomonadaceae (0.036 ± 0.022) declined substantially. Additionally, we also noted a decline in the order Cyanobacteriales, wherein at TP0 it had the highest abundance (0.421), whereas even in the early phase (excluding TP0) evident declines were observed (0.111 ± 0.061), which were only exacerbated in the later stages (middle [0.071 ± 0.042] and late [0.010 ± 0.011]).

*Viral composition*

Relative abundance of the virome was calculated separate from the microbiome. At TP0, the most abundant class was Megaviricites (0.42), which comprised 10 of the constructed vMAGs. Of these, 4 were annotated as Phycodnaviridae and 2 were annotated as Mimiviridae, both of which are algal infecting viruses. The abundance of Megaviricites declined as the experiment progressed, with marked declines even in the early phase 0.143 ± 0.181 (excluding TP0), and even larger declines in the middle (0.041 ± 0.028) and late (0.034 ± 0.025) phases [**Figure 1D**]. A total of 86 of the vMAGs were annotated as Caudoviricetes, which are bacteriophages. Caudoviricetes had low abundance at TP0 (0.17), but high abundance in the early (0.678 ± 0.296, excluding TP0), middle (0.792 ± 0.081), and late (0.711 ± 0.164) phases.

*Shifts in relative abundance in degradation associated MAGs.*

Additionally, for all DAMs, we also assessed TPM based abundance across phases which indicated stable levels of each MAG in the degradation experiment. TPM abundances for pMAG04 (in order of e4) 1.660 ± 1.133 (early), 4.716 ± 0.721(middle), and 2.268 ± 0.156 (late). In pMAG10, the TPM values were 0.828 ± 0.548, 3.039 ± 1.636, and 0.848 ± 0.419, respectively. pMAG18, had TPM values 2.523 ± 2.501, 4.265 ± 3.916, and 1.773 ± 1.671. Lastly, in pMAG23, the TPM values were 8.874 ± 17.288, 3.884 ± 7.718, and 47.408 ± 15.237. Therefore, the apparent shift in relative abundance appeared to be driven by a significant increase in pMAG23 abundance at the late phase, although overall, the remaining DAMs were still present in the microbiomes. Although shifts were observed, none were statistically significant.
