# Supplementary material for: Rafts of change: microbial and functional dynamics in simulated Sargassum strandings
Source: Appl Environ Microbiol. 2026 Mar 31;92(4):e02357-25. doi: 10.1128/aem.02357-25 (PMC13101531; doi:10.1128/aem.02357-25)
Supplement: Supplemental text 2 — Preliminary composition analysis. [file aem.02357-25-s0002.docx]

**Supplemental Text 2: Rafts of Change: Microbial and Functional Dynamics in Simulated *Sargassum* Strandings**

**Preliminary Composition Analysis**

*Summary of compositional analysis*

In addition to the multi-omic datasets, we also conducted a compositional analysis of total structural carbohydrates in degrading Sargassum filipendula (**Supplemental Table 8**). While this analysis revealed some biologically interesting trends, substantial variability among replicates limited our ability to draw robust biological conclusions for integration with the other omics datasets. As such, these results were not incorporated into the primary multi-omics scope of the main study. Nevertheless, we view this dataset as a valuable foundation for future work aimed at resolving the dynamics of structural carbohydrate turnover during Sargassum degradation and anticipate that it may serve as a useful resource for researchers pursuing complementary or more targeted experimental designs.

*Compositional analysis methods*

The *Sargassum* total structural carbohydrates compositional analysis method was based on a slightly modified protocol reported (1,2). Briefly, in two technical replicates for each of the four biological replicates, 0.025 g of *Sargassum* was weighed out and dried overnight at 40°C on an aluminum pan. After drying, the samples were then placed into glass tube with threaded tops and labelled accordingly. Next, 250 mL of 72% (w/w) sulfuric acid was added to each glass tube and placed in a hot water bath, at 30°C, for 1 hour with gentle vortexing every 10min. After 1h, 7 ml of deionized (DI) water was added to each tube, bringing the sulfuric acid concentration to 4% (w/w). After recapping, the tubes were placed upright and autoclaved for either 30min (for estimating total uronic acids) or 60min (for estimating total neutral sugars) at 121°C. After cooling, samples were filtered with a 0.2mm nylon filter and disposable syringe, to remove solids from seaweed hydrolysate supernatants, and placed in a vial for High Pressure Ion Chromatography (HPIC) analysis on a Dionex™ CarboPac™ PA10 IC Column in line with a Pulsed Amperometric Detector (PAD). The mobile phases consisted of a combined 1M sodium acetate and 200mM NaOH solution (for uronic acid separation), and a 200mM NaOH solution for neutral sugar separation. For the first 25min, the NaOH flowed at 0.4ml/min, with 1.2% NaOH and 98.8% ultrafiltered DI water flow composition. After that point, a shallow gradient was implemented to increase the 1M sodium acetate and 200mM NaOH in the flow composition for uronic acid separation, and then the column was regenerated and brought to the beginning flow composition comprised of 1.2% 200mM NaOH and ultrafiltered DI water. HPIC-PAD chromatogram peak integrations for monosaccharide standards were used to generate standard curves for individual sugars and quantify the concentration of monosaccharides in each seaweed hydrolysate. For uronic acids, 0.002 mg/ml – 0.06 mg/ml was used for the standard curves. For the neutral sugars, a range of 0.00025 mg/ml – 0.06 mg/ml standards. Here, based on an optimized acid hydrolysis protocol under development for *Sargassum*, the 30-minute hydrolysis method was used to quantify the uronic acids from less recalcitrant polysaccharides (e.g., alginate), while the 60min hydrolysis method was used for the neutral sugars from more recalcitrant polysaccharides (e.g., cellulose). This was based on the increased monosaccharide concentration observed for neutral sugars at 60 versus 30mins. Note that while the compositional method is still being optimized for more efficient analysis of *Sargassum* biomass, the relative composition trends reported in this study were found to be still valid.

Sargassum *compositional analysis results*

Based on the total structural carbohydrate compositional analysis conducted on the *Sargassum* samples from the *in vitro* degradation experiment at TP0 and TP9, two major trends were observed across four biological replicates on average; a significant decrease in mannitol released (*p*-value< 0.001), and a high variation in the guluronic acid released [**Supplemental Figure 4**]. Mannitol content in *Sargassum* from TP0 to TP9 decreased by nearly 50% likely due two reasons, metabolic uptake and accessibility. Mannitol can often act as a simple carbon source for the microbes actively growing on *Sargassum* and can be readily utilized. In addition, mannitol is also often chemically linked to the reducing end of polysaccharides in seaweed like laminarin that is further embedded within a matrix of crystalline cellulose found in the genus *Sargassum*. Therefore, glycosyl hydrolases active on cellulose and laminarin can further increase accessibility to mannitol during *in vitro* degradation of *Sargassum*. Aside from mannitol, wider variation at time points for TP0 and TP9 concentrations of guluronic acid were observed between various biological replicates. This suggests likely high variability in microbial activity/accessibility to the alginate and/or variability in alginate composition in the various *Sargassum* tissue samples tested in this study. Since we observed high variability in the uronic acids released, and not with the other monosaccharides, we suspect this is not an issue with the analytical protocol and is likely associated with the nature of the biological sample tested here. Additionally, we extracted proteins from the *Sargassum* tissues and found them to be active on the commercial alginate substrates further suggesting presence of alginate lyase activity that was higher in *Sargassum* TP9 samples versus the control TP0 (data not shown). Compositional analysis (and preliminary proteome activity analysis) supports the metagenomic findings by confirming the presence and activity of key CAZymes relevant to *Sargassum* deconstruction.

The concentration of all other structural carbohydrates-based monosaccharides such as arabinose, xylose, and mannuronic acid, remained largely unchanged between the timepoints sampled here. Important to note as well, there are statistically significant changes detected in some released sugars like fucose (*p*-value < 0.01), glucose (*p*-value < 0.05), and mannose (*p*-value < 0.001) between TP0 and TP9. This result could potentially reflect the enrichment of recalcitrant polysaccharides (like fucoidan) that are not readily degraded by microbial enzymes active on *Sargassum*. It is possible that structural polysaccharides are not showing a major change in relative composition for other reasons. Firstly, if there are specific polysaccharide structural linkages that are not being targeted by the upregulated genes and produced enzymes, the polysaccharide will not be efficiently degraded into its constituent monosaccharides. This is likely happening with fucoidan, as it can be highly complex and variable in its composition, requiring hundreds of enzymes, making it a difficult target for deconstruction (3). Second, the inaccessibility of polysaccharides within *Sargassum* due to structural arrangement of the cell wall and its recalcitrance to degradation could be another reason for the slow degradation detected. This likely makes it easier for microbial enzymes to target more readily accessible polysaccharides that are structurally less complex but can also impact the deconstruction of recalcitrant polysaccharides embedded within the tissue matrix, even if the enzyme is highly active. Finally, due to the inherent heterogeneity in biomass composition, given that polysaccharide abundance can vary throughout the different tissue types of *Sargassum* such as the thallus, and the sampling strategy there could be significant heterogeneity in the overall composition. A more refined sampling strategy and detailed material balance is needed to address some of these open questions.

In summary, composition analysis of the samples from TP0 and TP9 showed a significant decrease in mannitol, great variation in guluronic acid, and minimal to no change in other sugars. The neutral sugars and mannuronic acid underwent minimal change in their concentration between time points likely due to either the absence of effective enzymes relevant for substrate deconstruction, inaccessibility of the specific substrate, and/or due to lack of metabolic pathways in associated microbes to consume released sugars. Mannitol has likely decreased in concentration due to its use as a carbon source and deconstruction by enzymes active on laminarin. Lastly, guluronic acid showed a high deviation due to non-uniform microbial interaction. This data suggests that *Sargassum* targeting enzymes were present and would be beneficial for further isolation and examination in the broader deconstruction of the substrate.

**References**

1. Van Wychen, S.; Laurens, L. *Determination of Total Carbohydrates in Algal Biomass*. Nrel.gov. <https://docs.nrel.gov/docs/fy24osti/87500.pdf>.
2. Manns, D.; Deutschle, A. L.; Saake, B.; Meyer, A. S. Methodology for Quantitative Determination of the Carbohydrate Composition of Brown Seaweeds (Laminariaceae). *RSC Adv.* **2014**, *4* (49), 25736–25746. <https://doi.org/10.1039/C4RA03537B>.
3. Sichert, A.; Corzett, C. H.; Schechter, M. S.; Unfried, F.; Markert, S.; Becher, D.; Fernandez-Guerra, A.; Liebeke, M.; Schweder, T.; Polz, M. F.; Hehemann, J.-H. Verrucomicrobia Use Hundreds of Enzymes to Digest the Algal Polysaccharide Fucoidan. Nat. Microbiol. 2020, 5 (8), 1026–1039. <https://doi.org/10.1038/s41564-020-0720-2>.
