# Supplementary figures and images for: Rafts of change: microbial and functional dynamics in simulated Sargassum strandings
Source: Appl Environ Microbiol. 2026 Mar 31;92(4):e02357-25. doi: 10.1128/aem.02357-25 (PMC13101531; doi:10.1128/aem.02357-25)

# Beta Diversity without TP3

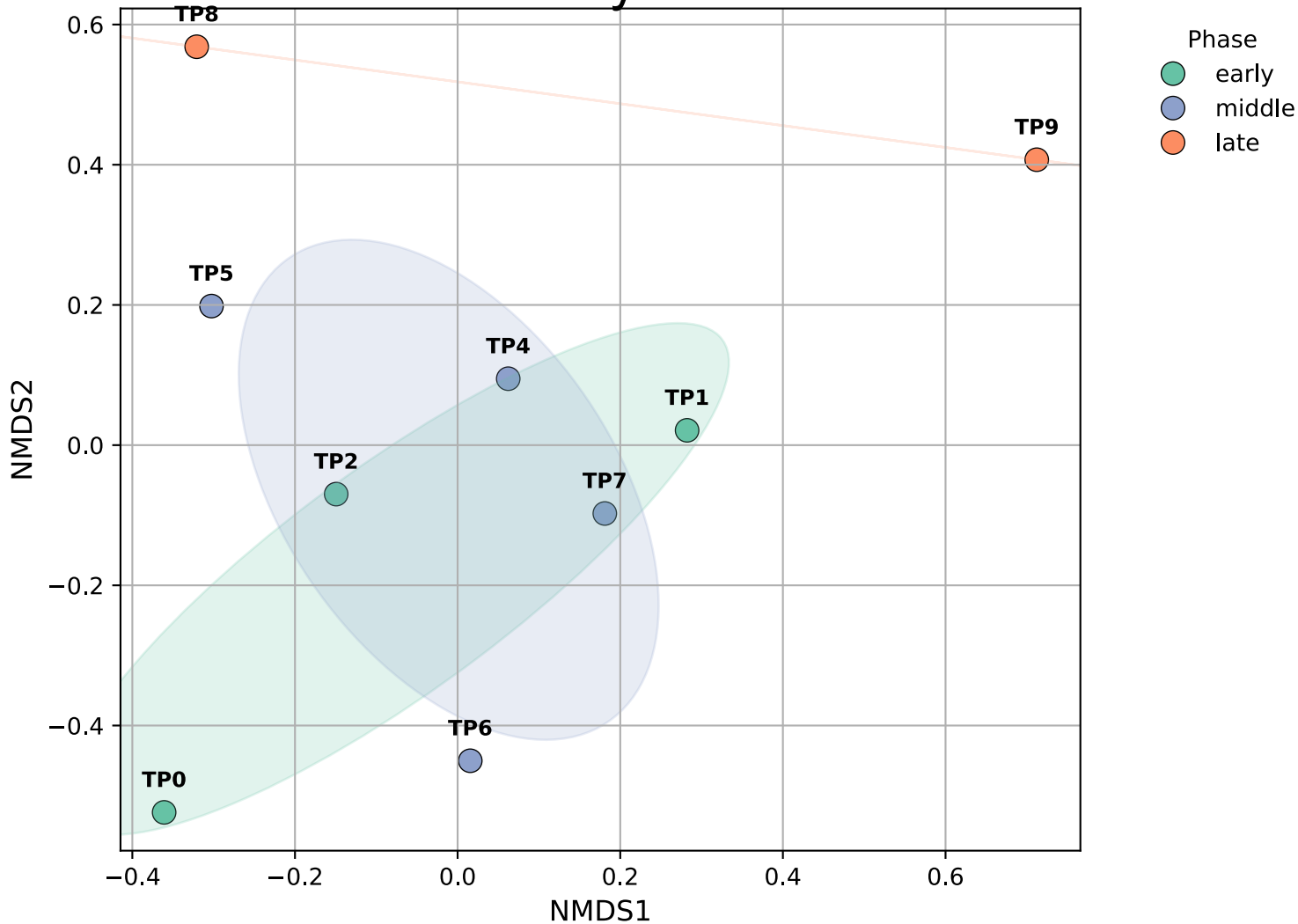

GSEA Phase Enrichment Summary

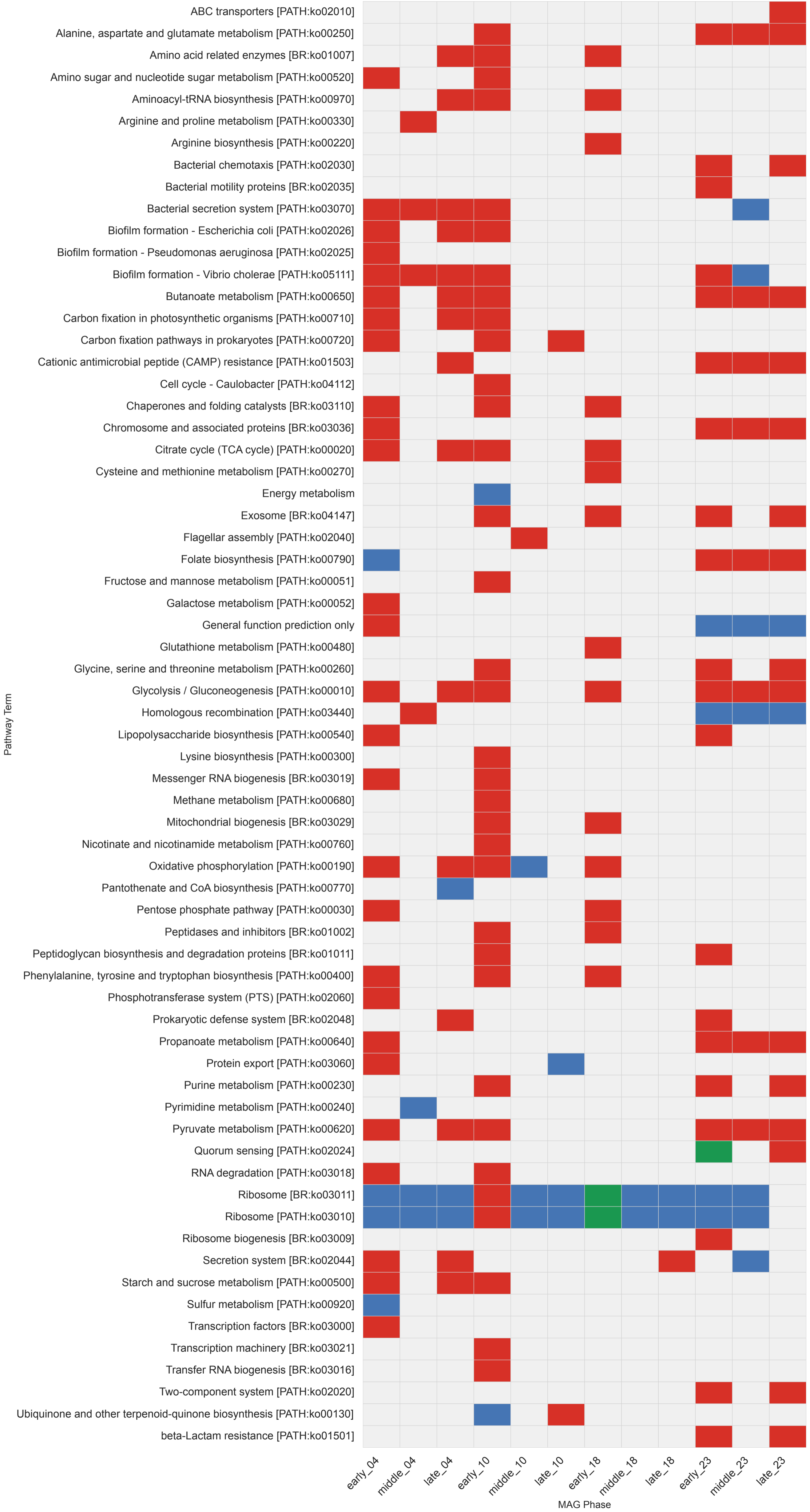



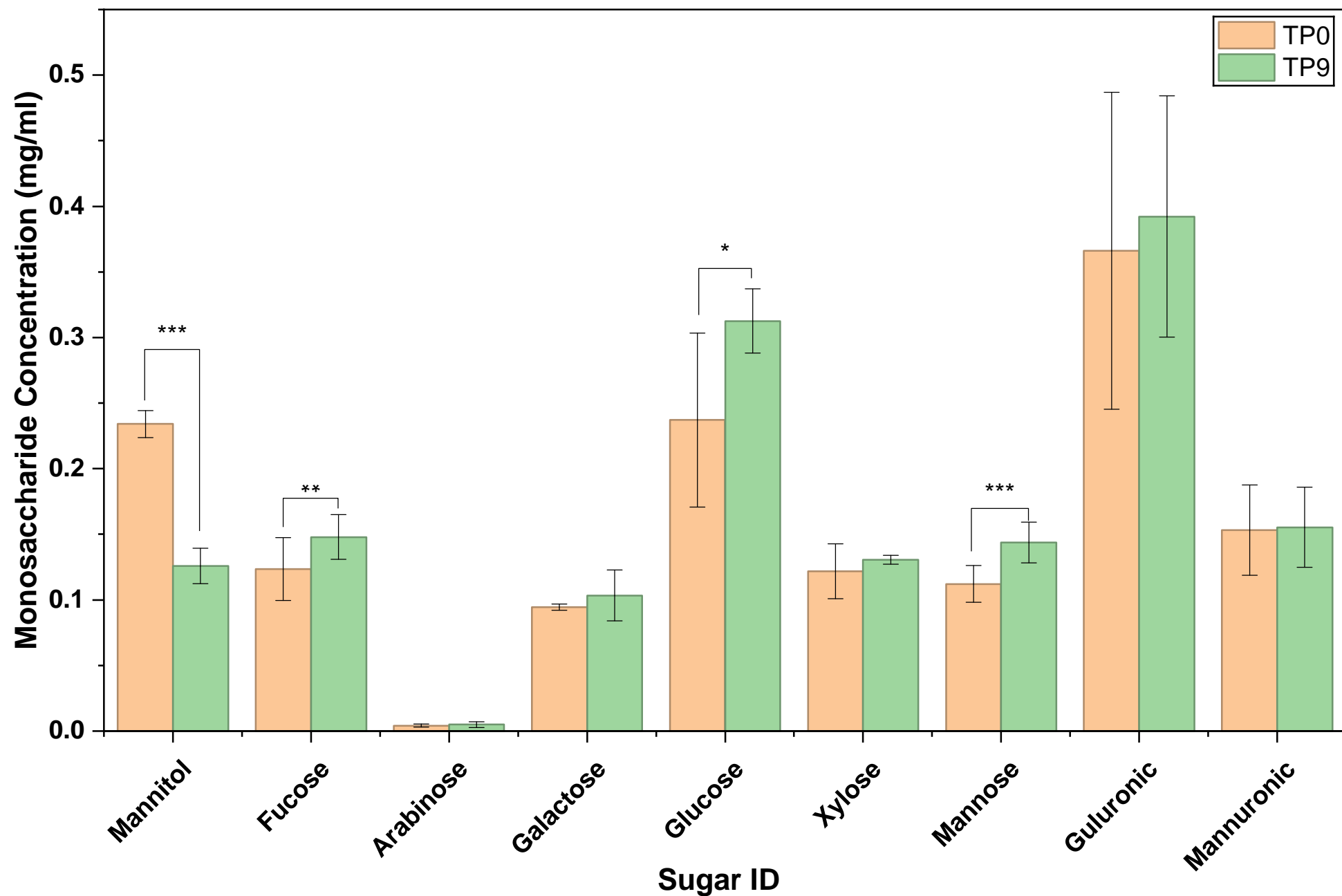

Supplement: Supplemental figures — Fig. S1 to S4. [file aem.02357-25-s0003.pdf]
