# Supplementary material for: Rafts of change: microbial and functional dynamics in simulated Sargassum strandings
Source: Appl Environ Microbiol. 2026 Mar 31;92(4):e02357-25. doi: 10.1128/aem.02357-25 (PMC13101531; doi:10.1128/aem.02357-25)
Supplement: Supplemental legends — Legends for all supplemental material. [file aem.02357-25-s0004.docx]

**Supplemental**

**Supplemental Table 1A:** Summary of all prokaryotic MAGs generated in this study.

**Supplemental Table 1B:** Summary of all viral MAGs generated in this study.

**Supplemental Table 1C:** Summary of all eukaryotic MAGs generated in this study.

**Supplemental Table 1D:** Summary of all plasmid MAGs generated in this study.

**Supplemental Table 2:** MAG abundance based on GPM that were subsequently used to calculate relative abundances.

**Supplemental Table 3:** All significant genes (binned and unbinned) as identified with pyDESeq2.

**Supplemental Table 4:** Gene set enrichment analysis results for the four degradation associated MAGs.

**Supplemental Table 5:** Arsenic detoxification genes as identified by BacMet2 database with known gene expression results.

**Supplemental Table 6A:** CAZymes annotated by dbCAN for each prokaryotic MAG

**Supplemental Table 6B:** Upregulated CAZymes associated with substrates of interest and phase in which gene is upregulated.

**Supplemental Table 6C:** dbCAN based CAZyme gene cluster annotation (CGC).

**Supplemental Table 7A:** Metagenomic read data.

**Supplemental Table 7B:** Meta-transcriptomic read data.

**Supplemental Table 8:** Preliminary results of composition analysis of *Sargassum*.

**Supplemental File 1:** Phylogenetic trees of arsenic detoxification genes in DAMs.

**Supplemental Figure 1:** Beta diversity (Aitchison distance) shown as an NMDS plot. Color scheme of points is shown in the legend**.** Timepoint 3 has been removed from this analysis. Individual timepoints have been presented over the corresponding dot. Ellipses have been drawn with a standard deviation of 3.0.

**Supplemental Figure 2:** A heatmap showing all pathways enriched in DAMs. Y-axis shows the pathway (based on C-level description KEGG), further grouped into general categories. The x-axis shows the pMAG and the respective phase. Red implies pathway is upregulated in the phase, blue implies pathway is downregulated in the phase, a green square implies a mixed response, i.e., enriched and downregulated in the same phase, and a grey square implies no change

**Supplemental Figure 3:** A heatmap showing distribution of substrate level annotated CAZyme gene clusters. The x-axis lists the annotated substrate, whereas the y-axis shows the pMAG, the fill indicates the number of CGCs within the pMAG that are annotated as the substrate.

**Supplemental Figure 4:** Bar graph detailing the carbohydrate compositional differences between TP0 and TP9 samples. The x-axis details the names of the individual monosaccharides, and the y-axis represents the normalized concentration (mg/ml) of each individual monosaccharide in the hydrolysate mixture (post-hydrolysis). The error bars represent the deviation in concentration between the samples for each respective timepoint (TP0 and TP9).

**Supplemental Text 1:** Additional results.

**Supplemental Text 2:** Preliminary composition analysis.
