# Supplementary material for: Rafts of change: microbial and functional dynamics in simulated Sargassum strandings
Source: Appl Environ Microbiol. 2026 Mar 31;92(4):e02357-25. doi: 10.1128/aem.02357-25 (PMC13101531; doi:10.1128/aem.02357-25)
Supplement: Supplemental file — Phylogenetic trees of arsenic detoxification genes in DAMs. [file aem.02357-25-s0005.pdf]

Supplemental File 1: Phylogenetic trees of arsenic detoxification genes in DAMs.

ArsA ; Upregulated

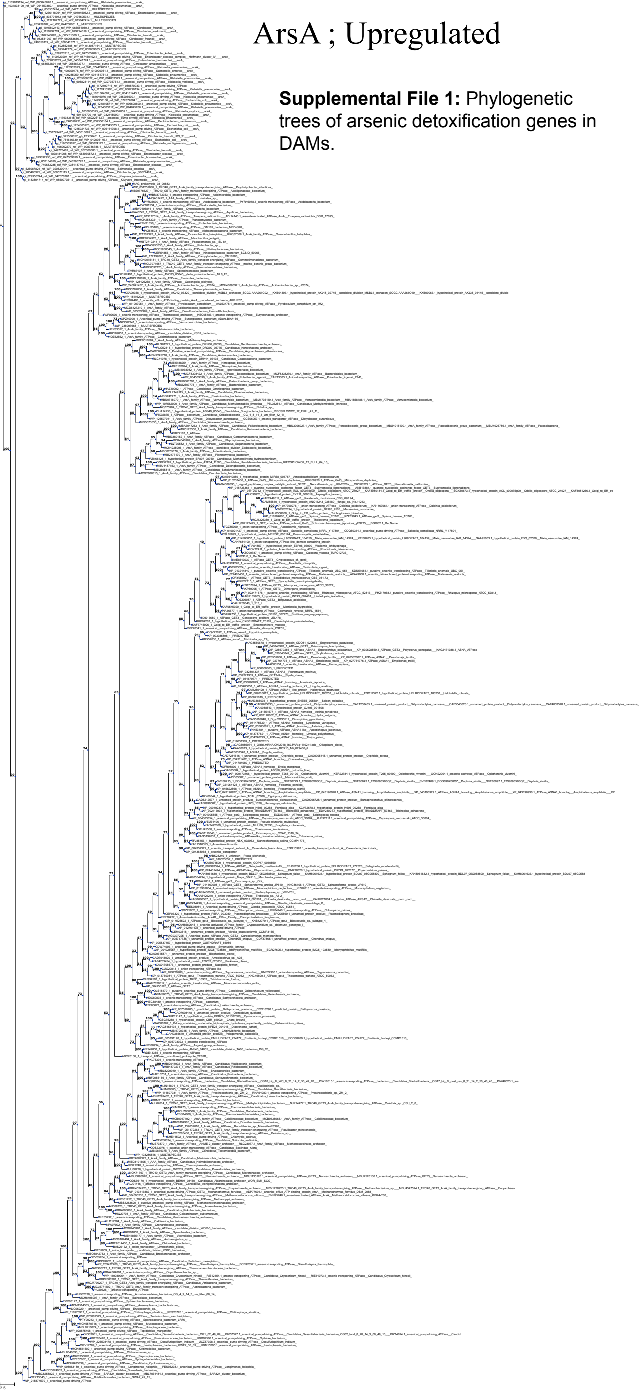

ArsA; Upregulated

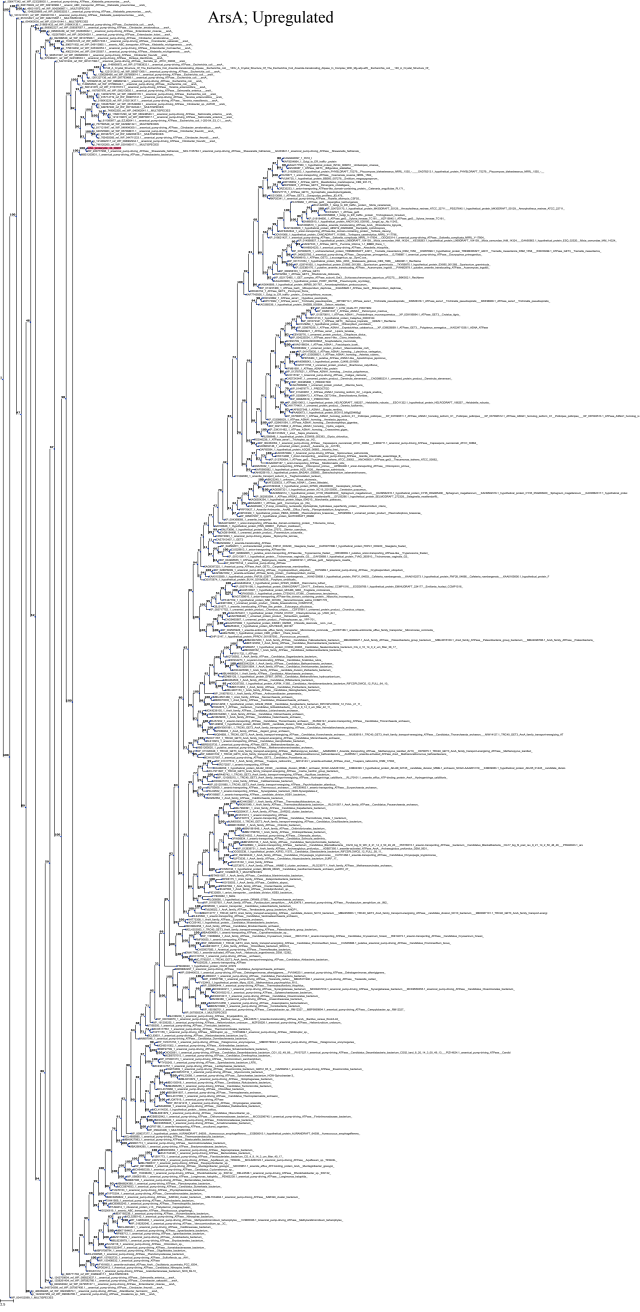

ArsD; Upregulated

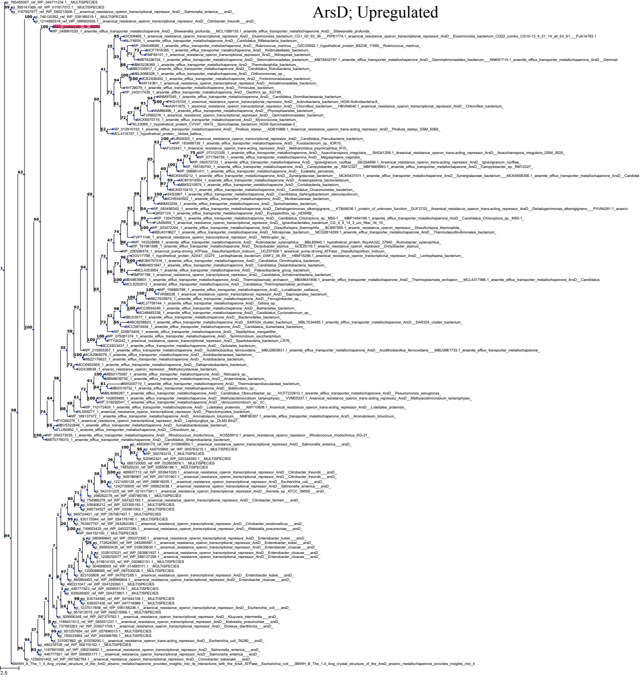

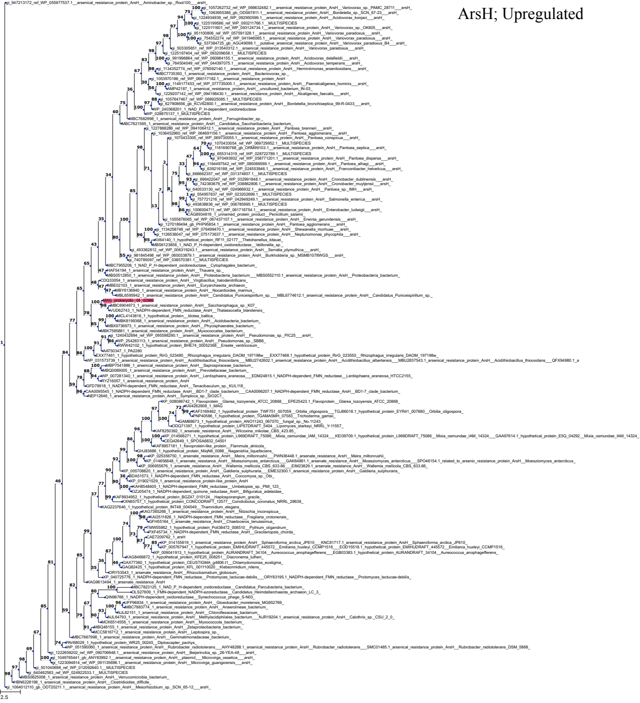
ArsH; Upregulated

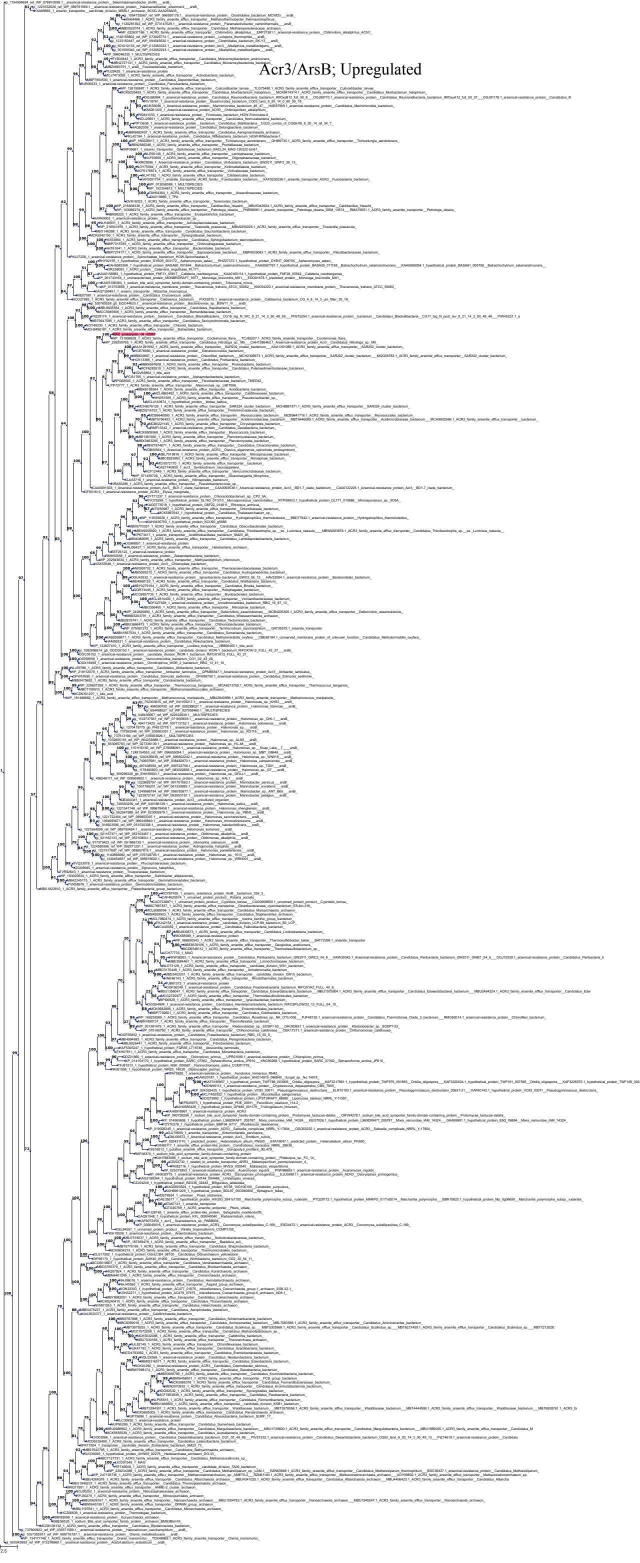

Acr3/ArsB; Upregulated
2.5

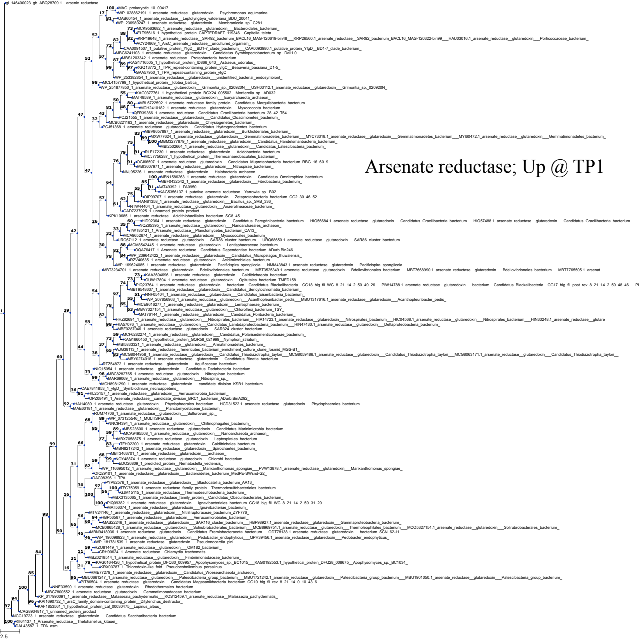
Arsenate reductase; Up @ TP1

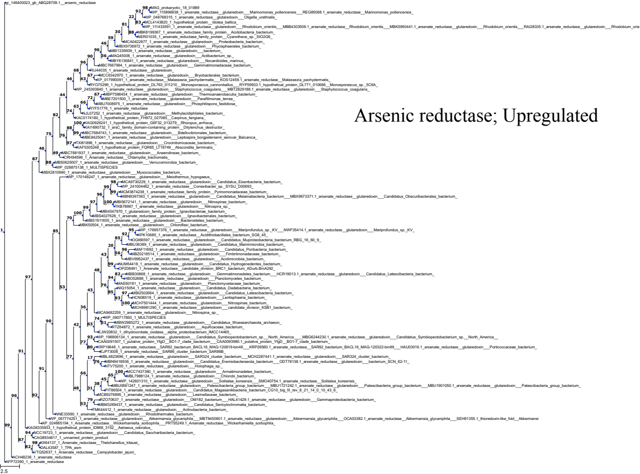
Arsenic reductase; Upregulated
2.5

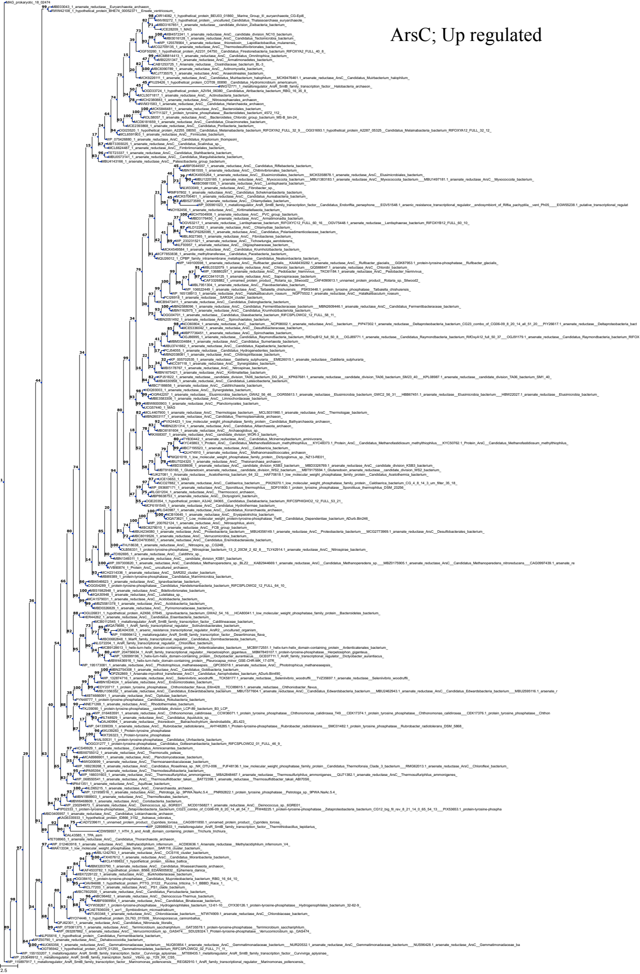
ArsC; Up regulated
2.5

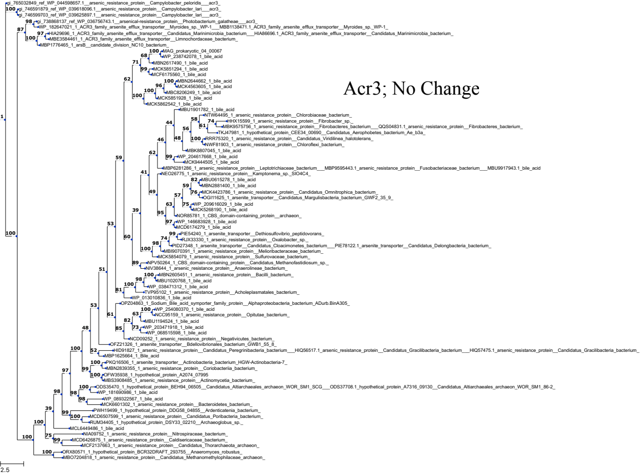
Acr3; No Change
gi_765032849_ref_WP_044598657.1__arsenic_resistance_protein__Campylobacter_peloridis___acr3_
gi_746591879_ref_WP_039618096.1__arsenic_resistance_protein__Campylobacter_lari___acr3_
gi_746599703_ref_WP_039625897.1__arsenic_resistance_protein__Campylobacter_lari___acr3_
gi_738868137_ref_WP_036756743.1__arsenical-resistance_protein__Photobacterium_galatheae___acr3_
WP_182647021_1_ACR3_family_arsenite_efflux_transporter__Myroides_sp._WP-1___MBB1138471.1_ACR3_family_arsenite_efflux_transporter__Myroides_sp._WP-1_
HIA29696_1_ACR3_family_arsenite_efflux_transporter__Candidatus_Marinimicrobia_bacterium___HIA86696.1_ACR3_family_arsenite_efflux_transporter__Candidatus_Marinimicrobia_bacterium_
MBE3584461_1_ACR3_family_arsenite_efflux_transporter__Limnochordaceae_bacterium_
MBP1776465_1_arsB__candidate_division_NC10_bacterium_
MAG_prokaryotic_04_00067
WP_238742078_1_bile_acid
MBN2617490_1_bile_acid
MCK5851294_1_bile_acid
MCF6175560_1_bile_acid
MBN2644662_1_bile_acid
MCK4563605_1_bile_acid
MBC8206249_1_bile_acid
MCK5851928_1_bile_acid
MCK5862542_1_bile_acid
MBU1901782_1_bile_acid
NTW64495_1_arsenic_resistance_protein__Chlorobiaceae_bacterium_
HHX15599_1_arsenic_resistance_protein__Fibrobacter_sp._
MBK9575756_1_arsenic_resistance_protein__Fibrobacteres_bacterium___QQS04831.1_arsenic_resistance_protein__Fibrobacteres_bacterium_
TKJ47981_1_hypothetical_protein_CEE34_00690__Candidatus_Aerophobetes_bacterium_Ae_b3a_
RRR75320_1_arsenic_resistance_protein__Candidatus_Viridilinea_halotolerans_
NWF81903_1_arsenic_resistance_protein__Chloroflexi_bacterium_
MBK8807045_1_bile_acid
WP_204617668_1_bile_acid
MCK9444505_1_bile_acid
MBP6281286_1_arsenic_resistance_protein__Leptotrichiaceae_bacterium___MBP9595443.1_arsenic_resistance_protein__Fusobacteriaceae_bacterium___MBU9917943.1_bile_acid
NEO26775_1_arsenic_resistance_protein__Kamptonema_sp._SIO4C4_
MBU0615278_1_bile_acid
MBN2881400_1_bile_acid
MCK4423786_1_arsenic_resistance_protein__Candidatus_Omnitrophica_bacterium_
OGI11625_1_arsenite_transporter__Candidatus_Margulisbacteria_bacterium_GWF2_35_9_
WP_209616029_1_bile_acid
MCK5268190_1_bile_acid
NOR85781_1_CBS_domain-containing_protein__archaeon_
WP_146683928_1_bile_acid
MCD6174279_1_bile_acid
PIE54240_1_arsenite_transporter__Dethiosulfovibrio_peptidovorans_
RJX33330_1_arsenic_resistance_protein__Oxalobacter_sp._
PID27348_1_arsenite_transporter__Candidatus_Cloacimonetes_bacterium___PIE78122.1_arsenite_transporter__Candidatus_Delongbacteria_bacterium_
MBI9070391_1_arsenic_resistance_protein__Melioribacteraceae_bacterium_
MCK5854079_1_arsenic_resistance_protein__Sulfurovaceae_bacterium_
NPV50264_1_CBS_domain-containing_protein__Candidatus_Methanofastidiosum_sp._
NIV38644_1_arsenic_resistance_protein__Anaerolineae_bacterium_
MBN2605451_1_arsenic_resistance_protein__Bacilli_bacterium_
MBU1020768_1_bile_acid
WP_038471312_1_bile_acid
TVP95102_1_arsenic_resistance_protein__Acholeplasmatales_bacterium_
WP_013010836_1_bile_acid
OPZ04863_1_Sodium_Bile_acid_symporter_family_protein__Alphaproteobacteria_bacterium_ADurb.BinA305_
WP_254080370_1_bile_acid
NCC95159_1_arsenic_resistance_protein__Opitutae_bacterium_
MBU1194524_1_bile_acid
WP_203471918_1_bile_acid
WP_068515598_1_bile_acid
NCD09252_1_arsenic_resistance_protein__Negativicutes_bacterium_
OFZ21326_1_arsenite_transporter__Bdellovibrionales_bacterium_GWB1_55_8_
HID91827_1_arsenic_resistance_protein__Candidatus_Peregrinibacteria_bacterium___HIQ56517.1_arsenic_resistance_protein__Candidatus_Gracilibacteria_bacterium___HIQ57475.1_arsenic_resistance_protein__Candidatus_Gracilibacteria_bacterium_
MBP1625664_1_Bile_acid
PKQ16506_1_arsenite_transporter__Actinobacteria_bacterium_HGW-Actinobacteria-7_
MBN2839355_1_arsenic_resistance_protein__Coriobacteriia_bacterium_
OFW35938_1_hypothetical_protein_A2074_07995
MBS3908485_1_arsenic_resistance_protein__Actinomycetia_bacterium_
ODS35470_1_hypothetical_protein_BEH94_06505__Candidatus_Altiarchaeales_archaeon_WOR_SM1_SCG___ODS37708.1_hypothetical_protein_A7316_09130__Candidatus_Altiarchaeales_archaeon_WOR_SM1_86-2_
WP_181690986_1_bile_acid
WP_089322567_1_bile_acid
MCK6601302_1_arsenic_resistance_protein__Bacteroidetes_bacterium_
PWH19499_1_hypothetical_protein_DDG58_04855__Ardenticatenia_bacterium_
MCD6507599_1_arsenic_resistance_protein__Candidatus_Poribacteria_bacterium_
RUM34405_1_hypothetical_protein_DSY33_02210__Archaeoglobus_sp._
MCL6449486_1_bile_acid
NIA09752_1_arsenic_resistance_protein__Nitrospiraceae_bacterium_
MCD6426875_1_arsenic_resistance_protein__Caldisericaceae_bacterium_
MCF2137663_1_arsenic_resistance_protein__Candidatus_Thorarchaeota_archaeon_
ORX80571_1_hypothetical_protein_BCR32DRAFT_293755__Anaeromyces_robustus_
MBO7204818_1_arsenic_resistance_protein__Candidatus_Methanomethylophilaceae_archaeon_
2.5

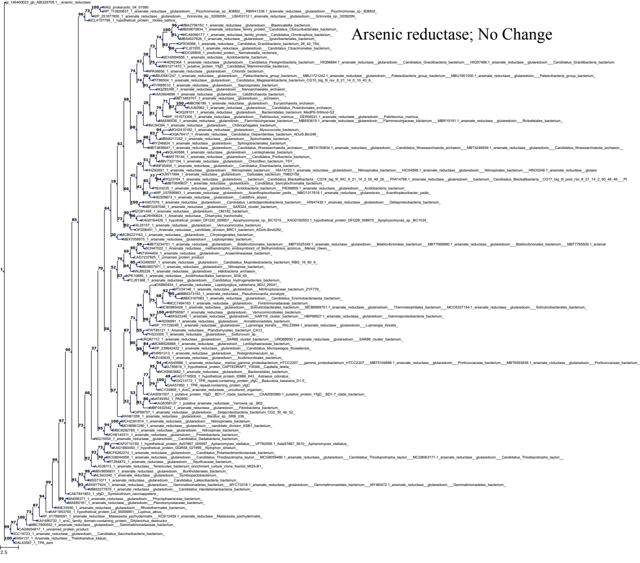

Arsenic reductase; No Change
2.5

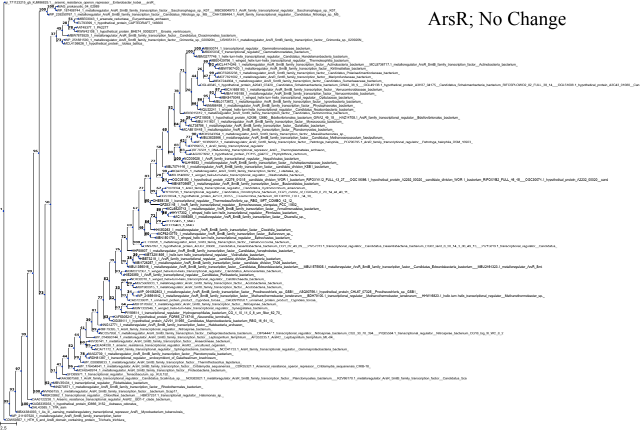
ArsR; No Change

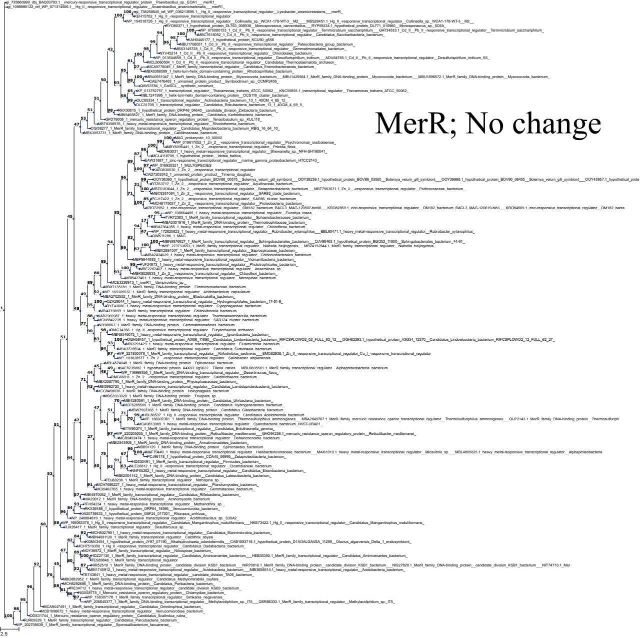
MerR; No change
2.5

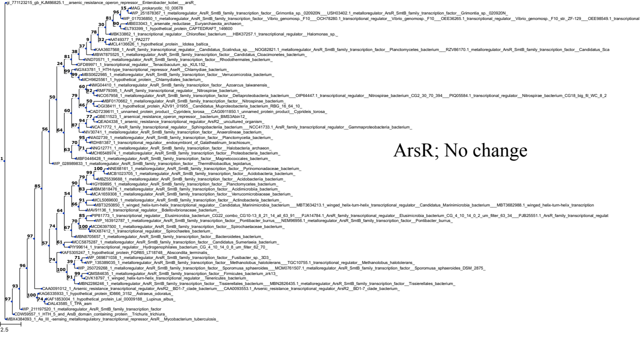

gi_771123215_gb_KJM86825.1__arsenic_resistance_operon_repressor__Enterobacter_kobei__arsR_
MAG_prokaryotic_10_00678
WP_251878367_1_metalloregulator_ArsR_SmtB_family_transcription_factor__Grimontia_sp._020920N___USH03402.1_metalloregulator_ArsR_SmtB_family_transcription_factor__Grimontia_sp._020920N_
WP_017036850_1_metalloregulator_ArsR_SmtB_family_transcription_factor__Vibrio_genomosp._F10___OCH78280.1_transcriptional_regulator__Vibrio_genomosp._F10___OEE36265.1_transcriptional_regulator__Vibrio_genomosp._F10_str._ZF-129___OEE98549.1_transcriptional
MBE03043_1_arsenate_reductase__Euryarchaeota_archaeon_
ELT93399_1_hypothetical_protein_CAPTEDRAFT_146600
MBK33862_1_transcriptional_regulator__Chloroflexi_bacterium___HBK37257.1_transcriptional_regulator__Halomonas_sp._
AAT49377_1_PA2277
MCL4136626_1_hypothetical_protein__Idotea_baltica_
KAA3607568_1_ArsR_family_transcriptional_regulator__Candidatus_Scalindua_sp.___NOG82821.1_metalloregulator_ArsR_SmtB_family_transcription_factor__Planctomycetes_bacterium___RZV86170.1_metalloregulator_ArsR_SmtB_family_transcription_factor__Candidatus_Sca
MBW7875525_1_metalloregulator_ArsR_SmtB_family_transcription_factor__Candidatus_Cloacimonetes_bacterium_
NND70571_1_metalloregulator_ArsR_SmtB_family_transcription_factor__Rhodothermales_bacterium_
GFD89971_1_transcriptional_regulator__Tenacibaculum_sp._KUL152_
NGX43781_1_HTH-type_transcriptional_repressor_AseR__Chlamydiae_bacterium_
MBS0622985_1_metalloregulator_ArsR_SmtB_family_transcription_factor__Verrucomicrobia_bacterium_
MCH9625561_1_hypothetical_protein__Chlamydiales_bacterium_
NMG04410_1_metalloregulator_ArsR_SmtB_family_transcription_factor__Azoarcus_taiwanensis_
RMF79395_1_ArsR_family_transcriptional_regulator__Nitrospirae_bacterium_
NCO57958_1_metalloregulator_ArsR_SmtB_family_transcription_factor__Deltaproteobacteria_bacterium___OIP64447.1_transcriptional_regulator__Nitrospirae_bacterium_CG2_30_70_394___PIQ05584.1_transcriptional_regulator__Nitrospirae_bacterium_CG18_big_fil_WC_8_2
MBF0170662_1_metalloregulator_ArsR_SmtB_family_transcription_factor__Nitrospinae_bacterium_
OGI38411_1_hypothetical_protein_A2V91_01955__Candidatus_Muproteobacteria_bacterium_RBG_16_64_10_
CAD7239611_1_unnamed_protein_product__Cyprideis_torosa___CAG0911850.1_unnamed_protein_product__Cyprideis_torosa_
GBE11523_1_arsenical_resistance_operon_repressor__bacterium_BMS3Abin12_
QEA04338_1_arsenic_resistance_transcriptional_regulator_ArsR2__uncultured_organism_
NCA71772_1_ArsR_family_transcriptional_regulator__Sphingobacteriia_bacterium___NCC41733.1_ArsR_family_transcriptional_regulator__Gammaproteobacteria_bacterium_
NIV30741_1_metalloregulator_ArsR_SmtB_family_transcription_factor__Anaerolineae_bacterium_
NIA02739_1_metalloregulator_ArsR_SmtB_family_transcription_factor__Planctomycetia_bacterium_
RDH81387_1_transcriptional_regulator__endosymbiont_of_Galathealinum_brachiosum_
NNG12771_1_metalloregulator_ArsR_SmtB_family_transcription_factor__Halobacteria_archaeon_
MCH6548974_1_metalloregulator_ArsR_SmtB_family_transcription_factor__Proteobacteria_bacterium_
MBF0446428_1_metalloregulator_ArsR_SmtB_family_transcription_factor__Magnetococcales_bacterium_
WP_028989833_1_metalloregulator_ArsR_SmtB_family_transcription_factor__Thermithiobacillus_tepidarius_
ArsR; No change
NNE68161_1_metalloregulator_ArsR_SmtB_family_transcription_factor__Pyrinomonadaceae_bacterium_
MCB1023705_1_metalloregulator_ArsR_SmtB_family_transcription_factor__Acidobacteria_bacterium_
MBZ5539688_1_metalloregulator_ArsR_SmtB_family_transcription_factor__Acidobacteriia_bacterium_
HGY89895_1_metalloregulator_ArsR_SmtB_family_transcription_factor__Planctomycetes_bacterium_
MBM3818476_1_metalloregulator_ArsR_SmtB_family_transcription_factor__Acidimicrobiia_bacterium_
MCA1659308_1_metalloregulator_ArsR_SmtB_family_transcription_factor__Verrucomicrobiaceae_bacterium_
MCL5069600_1_metalloregulator_ArsR_SmtB_family_transcription_factor__Actinobacteria_bacterium_
MBT3250850_1_winged_helix-turn-helix_transcriptional_regulator__Candidatus_Marinimicrobia_bacterium___MBT3634213.1_winged_helix-turn-helix_transcriptional_regulator__Candidatus_Marinimicrobia_bacterium___MBT3682988.1_winged_helix-turn-helix_transcription
MAV91136_1_transcriptional_regulator__Bdellovibrionaceae_bacterium_
PIP81773_1_transcriptional_regulator__Elusimicrobia_bacterium_CG22_combo_CG10-13_8_21_14_all_63_91___PJA14784.1_ArsR_family_transcriptional_regulator__Elusimicrobia_bacterium_CG_4_10_14_0_2_um_filter_63_34___PJB25551.1_ArsR_family_transcriptional_regulat
WP_163912787_1_metalloregulator_ArsR_SmtB_family_transcription_factor__Pontibacter_burrus___NEM96956.1_metalloregulator_ArsR_SmtB_family_transcription_factor__Pontibacter_burrus_
MCD6397500_1_metalloregulator_ArsR_SmtB_family_transcription_factor__Spirochaetaceae_bacterium_
RKX87412_1_transcriptional_regulator__Spirochaetes_bacterium_
MBN8705657_1_metalloregulator_ArsR_SmtB_family_transcription_factor__Bacteroidetes_bacterium_
MCC5875287_1_metalloregulator_ArsR_SmtB_family_transcription_factor__Candidatus_Sumerlaeia_bacterium_
PIY99614_1_transcriptional_regulator__Hydrogenophilales_bacterium_CG_4_10_14_0_8_um_filter_62_70_
KAF5305247_1_hypothetical_protein_FQR65_LT18748__Abscondita_terminalis_
WP_069871038_1_metalloregulator_ArsR_SmtB_family_transcription_factor__Fusibacter_sp._3D3_
WP_135389035_1_metalloregulator_ArsR_SmtB_family_transcription_factor__Methanolobus_halotolerans___TGC10755.1_transcriptional_regulator__Methanolobus_halotolerans_
WP_250729268_1_metalloregulator_ArsR_SmtB_family_transcription_factor__Sporomusa_sphaeroides___MCM0761507.1_metalloregulator_ArsR_SmtB_family_transcription_factor__Sporomusa_sphaeroides_DSM_2875_
QMS84635_1_metalloregulator_ArsR_SmtB_family_transcription_factor__Firmicutes_bacterium_zrk13_
QVK18797_1_winged_helix-turn-helix_transcriptional_regulator__Tenericutes_bacterium_
MBN2286246_1_metalloregulator_ArsR_SmtB_family_transcription_factor__Tissierellales_bacterium___MBN2826435.1_metalloregulator_ArsR_SmtB_family_transcription_factor__Tissierellales_bacterium_
CAA0091012_1_Arsenic_resistance_transcriptional_regulator_ArsR2__BD1-7_clade_bacterium___CAA0093553.1_Arsenic_resistance_transcriptional_regulator_ArsR2__BD1-7_clade_bacterium_
KAG6335933_1_hypothetical_protein_ID866_3152__Astraeus_odoratus_
KAF1853004_1_hypothetical_protein_Lal_00009188__Lupinus_albus_
DAL43585_1_TPA_asm
WP_211197520_1_metalloregulator_ArsR_SmtB_family_transcription_factor
CDW59557_1_HTH_5_and_ArsB_domain_containing_protein__Trichuris_trichiura_
MBX4384093_1_As_III_-sensing_metalloregulatory_transcriptional_repressor_ArsR__Mycobacterium_tuberculosis_
2.5

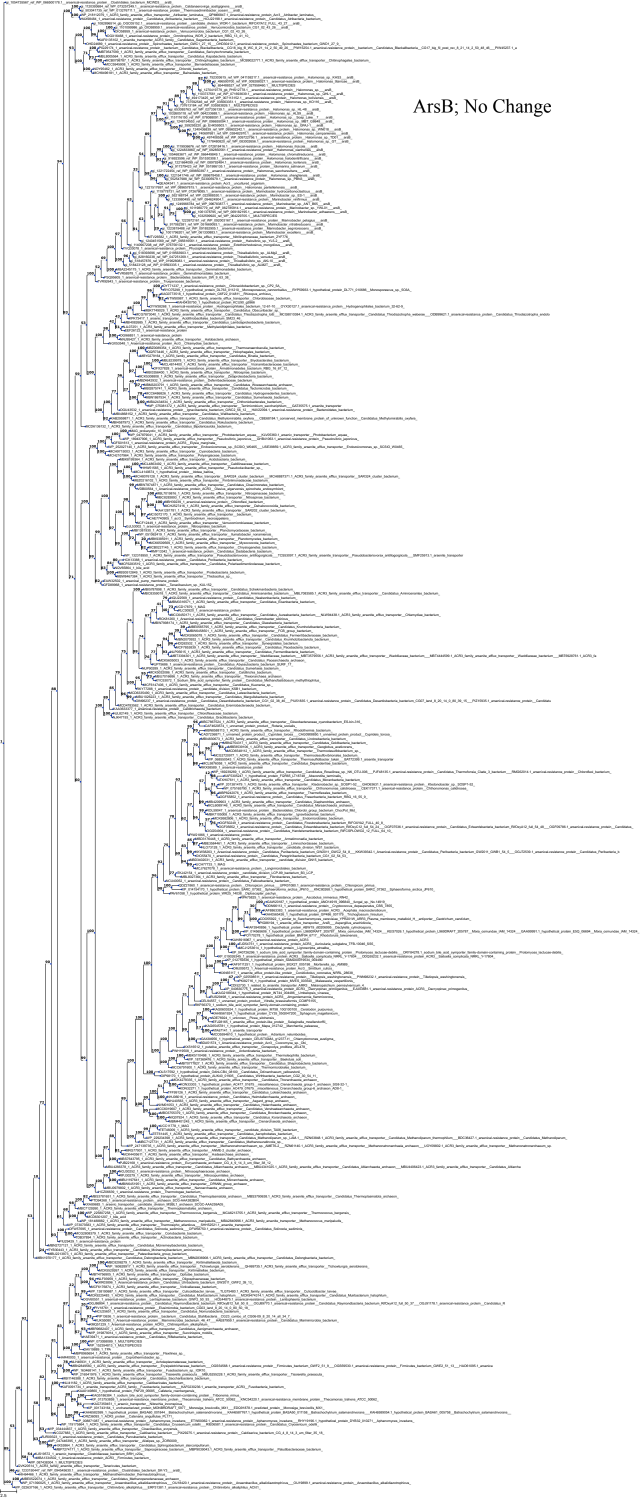
ArsB; No Change

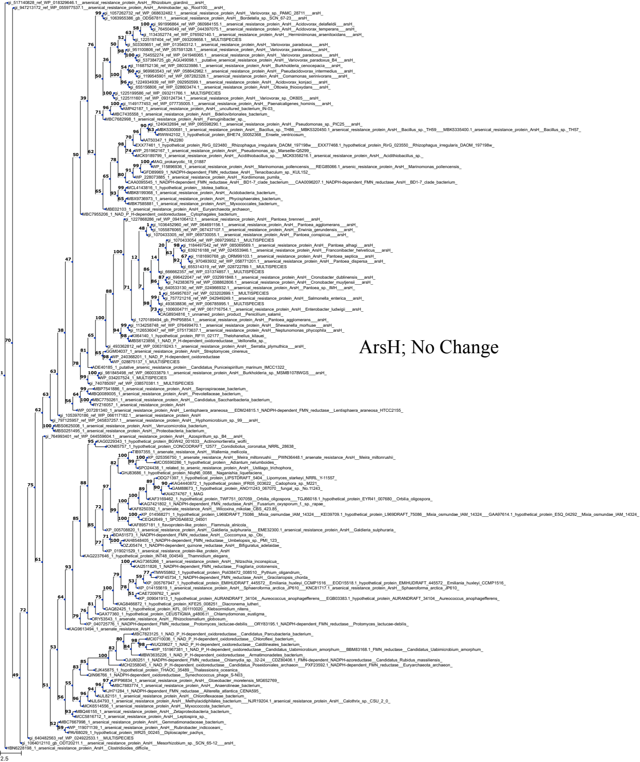
ArsH; No Change
2.5

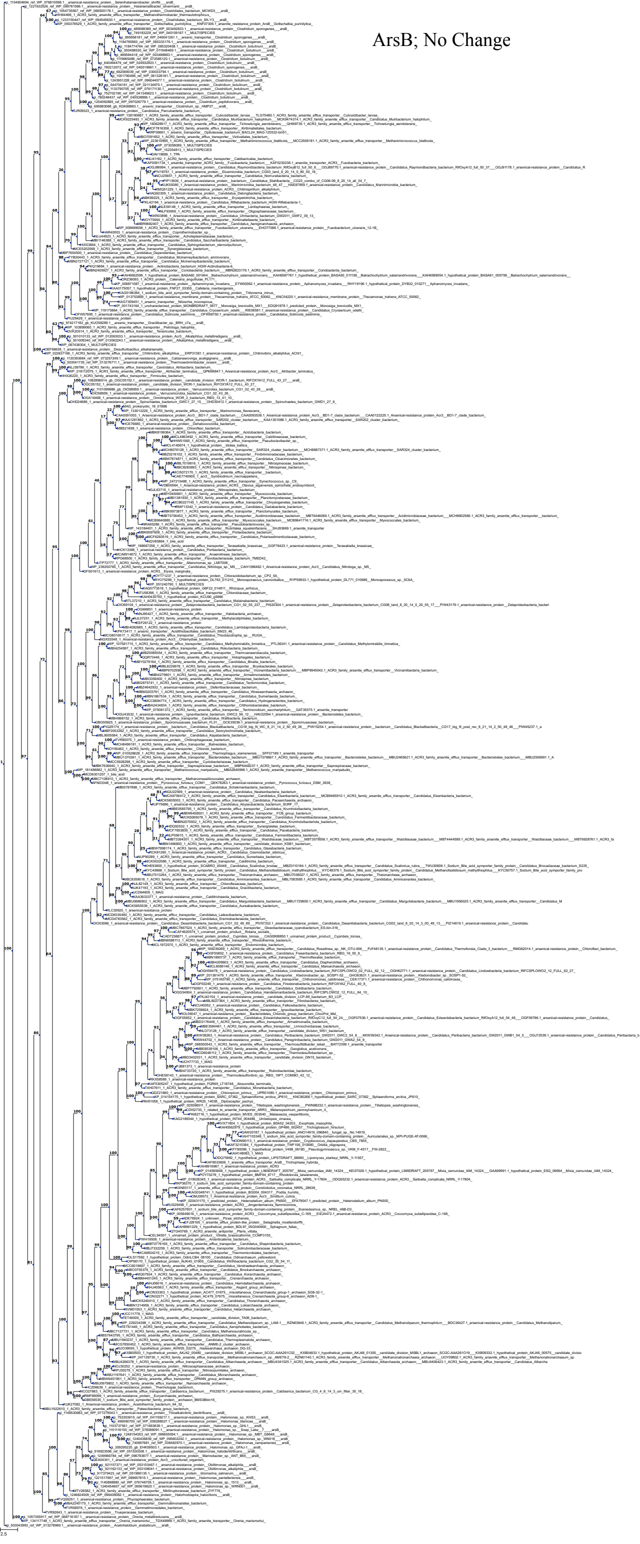
ArsB; No Change
2.5

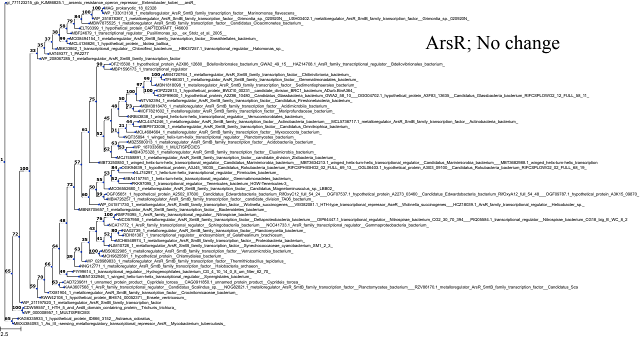
ArsR; No change
2.5

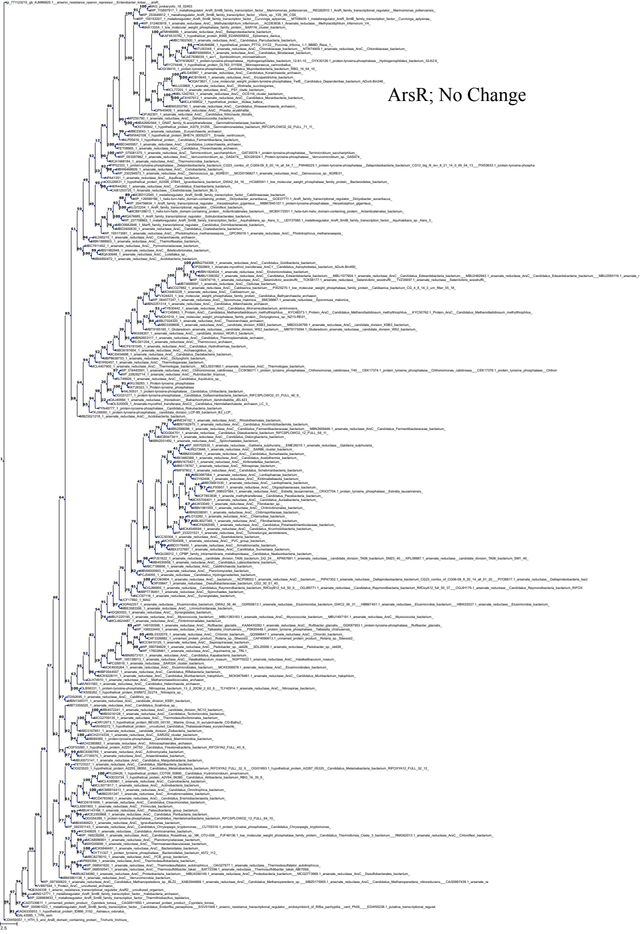
ArsR; No Change

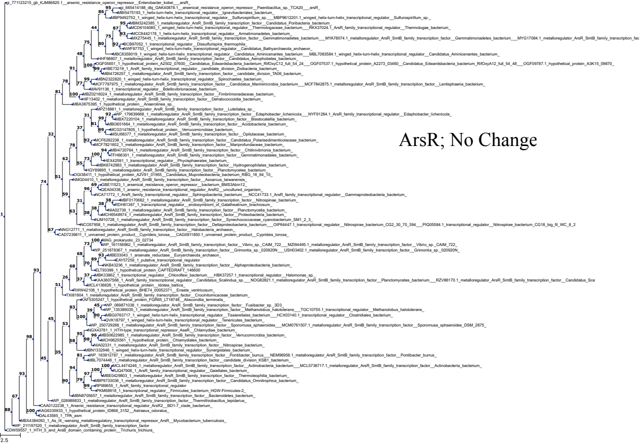
ArsR; No Change
2.5
